# The impact of dose rate on responses of human lens epithelial cells to ionizing irradiation

**DOI:** 10.1038/s41598-024-62679-8

**Published:** 2024-05-28

**Authors:** Yusuke Matsuya, Tatsuhiko Sato, Yoshie Yachi, Hiroyuki Date, Nobuyuki Hamada

**Affiliations:** 1 Faculty of Health Sciences, Hokkaido University, Kita-12 Nishi-5, Kita-ku, Sapporo, 060-0812 Japan; 2 Research Group for Radiation Transport Analysis, Nuclear Science and Engineering Center, Japan Atomic Energy Agency (JAEA), 2-4 Shirakata, Tokai, Ibaraki 319-1195 Japan; 3 Graduate School of Health Sciences, Hokkaido University, Kita-12 Nishi-5, Kita-ku, Sapporo, Hokkaido 060-0812 Japan; 4 Biology and Environmental Chemistry Division, Sustainable System Research Laboratory, Central Research Institute of Electric Power Industry (CRIEPI), Chiba, 270-1194 Japan

**Keywords:** Lens epithelial cells, DNA double-strand breaks, Surviving fraction, Dose-rate effects, Cell death, Biophysics

## Abstract

The knowledge on responses of human lens epithelial cells (HLECs) to ionizing radiation exposure is important to understand mechanisms of radiation cataracts that are of concern in the field of radiation protection and radiation therapy. However, biological effects in HLECs following protracted exposure have not yet fully been explored. Here, we investigated the temporal kinetics of γ-H2AX foci as a marker for DNA double-strand breaks (DSBs) and cell survival in HLECs after exposure to photon beams at various dose rates (i.e., 150 kVp X-rays at 1.82, 0.1, and 0.033 Gy/min, and ^137^Cs γ-rays at 0.00461 Gy/min (27.7 cGy/h) and 0.00081 Gy/min (4.9 cGy/h)), compared to those in human lung fibroblasts (WI-38). In parallel, we quantified the recovery for DSBs and cell survival using a biophysical model. The study revealed that HLECs have a lower DSB repair rate than WI-38 cells. There is no significant impact of dose rate on cell survival in both cell lines in the dose-rate range of 0.033–1.82 Gy/min. In contrast, the experimental residual γ-H2AX foci showed inverse dose rate effects (IDREs) compared to the model prediction, highlighting the importance of the IDREs in evaluating radiation effects on the ocular lens.

## Introduction

A cataract is a cloudy area in the normally transparent crystalline lens of the eye, leading to vision impairment. The International Commission on Radiological Protection (ICRP) listed cataracts as a radiation hazard in 1950^1^, and has recommended dose limits since 1954^2^. ICRP defined a threshold-type dose response in 1969^3^, and classified cataracts as deterministic effects in 1990^4^ and then tissue reaction in 2007^5^. ICRP has revised dose limits several times and recommended a reduction in the dose limit in 2011^6,7^. Such changes in dose limits were primarily based on epidemiological evidence^7–9^, and radiation responses of the lens and the mechanisms behind radiation cataracts remain incompletely understood. In the latest recommendation, ICRP assumes the same dose threshold of 0.5 Gy for cataracts following acute, fractionated/protracted, or chronic exposures^10^, while recognizing uncertainty in judging such no dose rate effect for radiation cataractogenesis^7^.

In the eye, lens epithelial cells (LECs) are organized as a monolayer that covers the anterior surface of the lens, and play an important role in the lens function^14^. Thus, in evaluating the biological responses of lens, it is essential to study the responses of the LECs. In 2014, Fujimichi and Hamada reported that clonogenic survival of primary human LECs (HLECs) is comparable to that of WI-38 primary human lung fibroblasts, suggesting that cell killing may not underlie high radiosensitivity of the lens^15^. In 2017, Hamada analyzed the temporal kinetics of the p53-binding protein 1 (53BP1) foci as a marker for repair of DNA double-strand breaks (DSBs), after acute irradiation at 0.42–0.45 Gy/min, and found that a growth delay in a subset of the HLECs due most likely to unrepaired or incompletely repaired DSBs, but without changing cell viability^16^. Against radiation-induced DSBs, non-homologous end joining and homologous recombination seem to function in LECs, which have been observed by both pathways of 53BP1 and Rad51^17^. The dose–response for the DNA damage in LECs has been reported to show nonlinear in the dose range below 1.0 Gy^17^. Barnard et al. investigated the residual 53BP1 foci in the murine lens at various dose rates of 0.014–0.3 Gy/min, showing inverse dose rate effects (IDREs where the lower the dose rate the higher the biological effectiveness)^18^. In 2021, Ahmadi et al. explored the early responses (such as cell viability and the residual 53BP1 foci) to low doses at 0.065 and 0.3 Gy/min using the immortalized HLECs (HLE-B3 cells) and the primary HLECs^18^. The potential impact of DNA damage on cataractogenesis in the lens has been documented^17,19^, and oxidative DNA damage seems to remain in the lens epithelium at 72 h post-irradiation^20^. As such, there is a growing body of experimental evidence for dose and dose-rate responses on DNA damage, but more quantitative data on dose rate dependence would be needed.

For a quantitative evaluation to clarify the mechanism of the cellular responses of HLECs, combination of an experimental study and a theoretical study using a biophysical model would be useful. Among various mathematical models for analyzing in vitro data, an integrated microdosimetric-kinetic (IMK) model^21^ that considers DNA damage repair during and after irradiation^22,23^ has the advantage of predicting the sparing effects of DNA damage responses and cell killing under fractionated/protracted exposures based on the experimental DSB repair rate. Meanwhile, Sakashita et al*.* proposed the first biophysical model for human cataractogenesis that allows the prediction of a relationship between radiation dose and cataract onset at various ages^24^, with the model parameters (e.g., DNA damage and repair) optimized to reproduce the Beaver Dam Eye Study data^25^. For more developments, a mechanistic model with the model parameters based on actually measured biological data would be required, and in this regard, the IMK model analysis using in vitro HLEC data is a powerful approach for the study on dose-rate effects.

In this study, we first obtained the experimental data for the temporal kinetics of nuclear DSB and cell survival data in HLECs after exposure to photon beams at five different dose rates in a range between 0.00081 and 1.82 Gy/min. Compared to the responses of the different normal human cell line (WI-38), the experimental results coupled with the IMK model quantitatively present dose-rate dependence for DSB repair and cell survival in HELCs. With this approach, we here report the IDREs on residual DSB in HLECs.

## Materials and methods for biological experiments

### Cell culture

We used two normal human cell lines, HLECs human lens epithelial cells (SCR-6550, ScienCell Res. Lab., Carlsbad, CA) and WI-38 primary lung fibroblasts (RCB0702, RIKEN, Tokyo, Japan). HLECs were derived from a 20-week gestation male as a donor, and grown in EpiCM (epithelial cell culture medium containing 2% fetal bovine serum (FBS) and 1% growth supplement) (SCR-4101, ScienCell Res. Lab., Carlsbad, CA) in 75 cm^2^ tissue culture (T75) flasks precoated with poly-L-lysine (SCR-4013, ScienCell Res. Lab., Carlsbad, CA), as described^15^. WI-38 cells were grown in Dulbecco’s modified Eagle’s medium/nutrient mixture F-12 (DMEM/F12) (D8437, Sigma, Kawasaki, Japan) supplemented with 10% FBS (Nichirei Bioscience Inc., Tokyo, Japan), as described^26^. These cell lines were maintained at 37 °C in a humidified atmosphere of 5% CO_2_. Prior to irradiation, the cells were seeded onto the *ϕ*12-mm glass-based dishes (3911–035, IWAKI) for DSB measurement and on the T25 flasks for survival measurement. The numbers of cells plated onto the T25 flask for survival assay were 1 × 10^3^, 3 × 10^3^, 5 × 10^3^, and 1 × 10^4^ for 0 Gy, 2 and 4 Gy, 6 Gy, and 10 Gy, respectively. On the other hand, the sufficient number of cells (about 5 × 10^5^ for WI-38 cells and about 1 × 10^5^ for HLEC) was seeded onto the glass-based dishes to be in the semiconfluent condition. Before irradiation, the cells were allowed to adhere at least overnight. The cell-cycle distributions prior to irradiation for both cell lines used in this study are shown in Fig. S1.

### Irradiation

**Figure 1:**
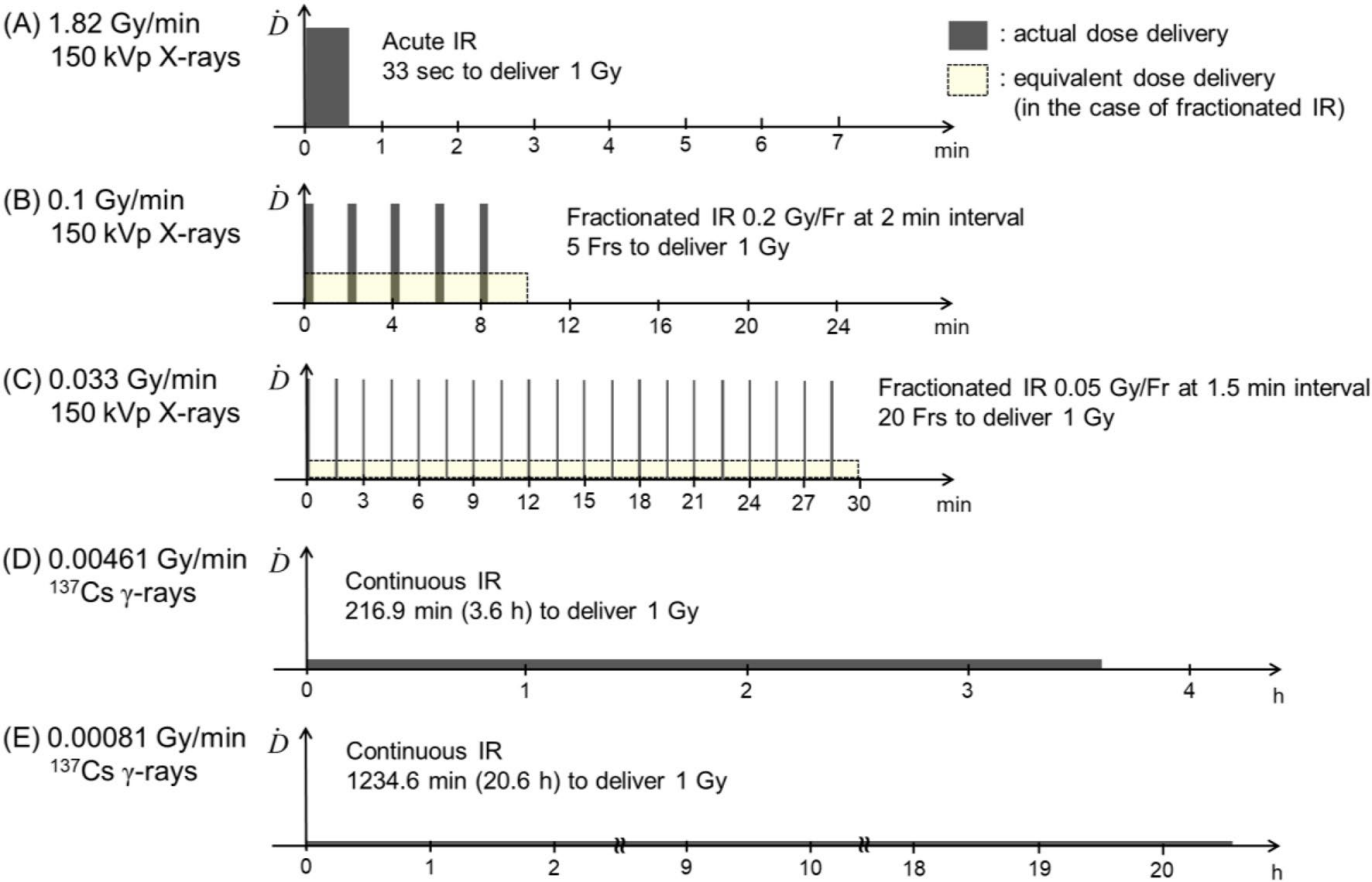
Illustration for dose delivery at various dose rates in this study. (**A**) 1.82 Gy/min by single irradiation with 150 kVp X-rays, (**B**) 0.1 Gy/min in fractions with 150 kVp X-rays, (**C**) 0.033 Gy/min in fractions with 150 kVp X-rays, (**D**) 0.00461 Gy/min (27.7 cGy/h) by continuous irradiation with ^137^Cs γ-rays, and (**E**) 0.00081 Gy/min (4.9 cGy/h) by continuous irradiation with ^137^Cs γ-rays. Fr fraction, IR ionizing irradiation.

The cells were exposed at room temperature to 150 kVp X-rays (MBR-1520R-4, Hitachi Medical Co., Tokyo, Japan) or ^137^Cs γ-rays. The dose rate of 150 kVp X-rays (1-mm Al filtration, a source surface distance of 550 mm) used in this study was 1.82 Gy/min, the radiation field of which was evaluated^27^. The air kerma was monitored with a thimble ionization chamber (HSY-1001, Hitachi Medical Co., Tokyo, Japan). The field size is *ϕ*400 mm, which is sufficiently bigger than the culture flask and dish. Using the fractionation regimens comprised of 0.2 Gy/fraction (Fr) (with 6.59 s dose-delivery time) at 120 s (2 min) intervals and 0.05 Gy/Fr (with 1.65 s dose-delivery time) at 90 s (1.5 min) intervals, we made the average dose rates of 0.1 and 0.033 Gy/min, respectively. The dose delivery was controlled to be stopped when the doses reached 0.2 and 0.05 Gy by the dosimeter attached to the X-ray irradiator. The dose rate of ^137^Cs γ-rays in air (i.e., air kerma rate) was measured using an ionization chamber (NE 2571, Nuclear Enterprises Technology Ltd., Reading, UK), and converted to that in water according to the International Atomic Energy Agency (IAEA) Technical Report Series No. 277^28^. The radiation field was evaluated using a general-purpose Monte Carlo code for radiation transport, i.e., Particle and Heavy Ion Transport code System (PHITS)^29^, as described^26^. According to the inverse square law of distance, the γ-ray dose-rate was changed to 0.00461 Gy/min (27.7 cGy/h) (at 5 mm from a ^137^Cs source) and 0.00081 Gy/min (4.9 cGy/h) (at 15 mm from a ^137^Cs source). Note that the dose flatness (dose variation < a few %) in the measuring area of γ-H2AX focus formation assay (i.e., *ϕ*6 mm area at the center of the glass-based dish) was also confirmed using the PHITS code. The irradiation schemes for five different dose rates (1.82, 0.1, 0.033 Gy/min, 0.00461 Gy/min (27.7 cGy/h) and 0.00081 Gy/min (4.9 cGy/h)) used in this study are depicted in Fig. 1. When irradiating the cells, cell culture medium in the T25 flask and 12-mm glass-based dishes was approximately 2 mm thick.

### γ-H2AX focus formation assay

The dynamics of DSBs in HLECs and WI-38 cells were measured with the phosphorylated histone H2AX (γ-H2AX) focus formation assay, as described^26^. Rothkamm et al*.* have reported that the number of γ-H2AX foci decreases rapidly to about 50% of the initial level within 1 h with increasing time after acute irradiation^30^, suggesting that γ-H2AX is suitable for detecting early DSB. We therefore selected γ-H2AX as a DSB marker. To evaluate the repair dynamics, the cells were fixed in 4% paraformaldehyde on ice for 10 min at 0.5, 1, 2.5, 6, 24, and 48 h after acute exposure at 1.82 Gy/min. To evaluate dose-rate effects, the cells were also fixed at 0.5 and 1 h after the end of irradiation and 24 and 48 h after the start of irradiation at five different dose rates. In general, “after irradiation” means “after the end of irradiation.” Because time is important in interpreting the results of DSBs, we explicitly specify the timepoints for detection of DSBs (i.e., after the start/end of irradiation).

After fixation, the cells were rinsed with phosphate buffered saline (PBS) and permeabilized in 0.2% Triton X-100 in PBS for 5 min. The cells were also blocked in 1% bovine serum albumin (BSA) in PBS for 30 min. After that, the cells were incubated at 4 °C overnight with a primary antibody against γ-H2AX (ab26350, Abcam) diluted 1:400 by 1% BSA in PBS, and rinsed with 1% BSA in PBS three times. The cells were then incubated for 2 h in the dark at room temperature with Alexa Fluor 594-conjugated goat-anti-mouse IgG (ab150116, Abcam) diluted 1:250 by 1% BSA in PBS. After three washes with 1% BSA in PBS, cell nuclei were counterstained with 1 μg/ml DAPI (62248, Thermo Fisher Scientific) for 15 min. After a rinse in methanol, we detected γ-H2AX foci using a fluorescent microscope (model BZ-9000; Keyence, Osaka, Japan), and counted the number of nuclear foci using Image J^31,32^. The experiment was repeated at least two times for each cell line, and the analysis was performed using about 100 cells.

### Clonogenic survival assay

Cell survival was measured using clonogenic assay as described^26^. The cells were counted with a hemocytometer (Erma, Tokyo, Japan), plated in T25 flasks (156367, Nunc, Waltham, MA, USA), and allowed to adhere overnight before irradiation. The irradiated cells were incubated for 14 days before staining with 0.5% crystal violet in 70% methanol. The surviving fraction was calculated as the ratio of the plating efficiency of the irradiated group to that of the non-irradiated group.

### Statistics

Scheffe's F test was used for nuclear γ-H2AX foci because the distribution of nuclear γ-H2AX foci does not follow the normal distribution and homoscedasticity^33^. Meanwhile, a paired t-test was used for clonogenic survival assuming the normal distribution and homoscedasticity based on our previous report^27^. Less than 5% was considered significant. Statistical tests were performed with Statcell4^34^.

## Model overview and analysis

To quantitatively discuss the measured DSB and survival data, we performed a theoretical analysis by using an IMK biophysical model^21,35^. The IMK model considers various factors (e.g., microdosimetry, DNA repair kinetics, cell cycle phase, oxygen effects, intercellular communication^36^, and existence of cancer stem-like cells^37^) that affect biological effects. Here, we used the IMK model considering DNA repair kinetics during and after irradiation to evaluate the dose-rate dependence (in other words, the impact of DNA repair on biological effects). Provided below is an overview of the IMK model used in this study.

### Assumption in the IMK model

We assume that the cell nucleus is subdivided into multiple micron-order territories (so-called domains) to incorporate microdosimetry defined in International Commission on Radiation Units and Measurements (ICRU) Report 36^38^. The domain shape is for simplicity defined as a sphere with a radius of 0.5–1.0 μm^39,40^, which can be measured by the tissue equivalent proportional counter^41^ or estimated by Monte Carlo track-structure simulation^42^. The cytotoxic, but reparable DNA lesions are defined as potentially lethal lesions (PLLs). During irradiation, the PLL is induced in a domain packaging DNA amount of *g* (kg) with energy deposition (J) per kg of domain (so-called specific energy defined in ICRU Report 36^38^) *z* (Gy)^21^. The PLL can gradually transform into non-reparable lesions defined as lethal lesions (LLs) or all be repaired with no PLL remaining as below:

(i) A first-order process by which a PLL may transform into an LL at a constant rate *a* (h^−1^);
(ii) A second-order process by which two PLLs may interact and transform into the LL at a constant rate *b*_d_ (h^−1^);
(iii) A first-order process by which the PLL may be repaired at a constant rate *c* (h^−1^).

The domains may be interpreted as interphase chromosome territories^43^, while PLL and LL may be associated with DSBs^36,43^.

## DNA lesion kinetics during and after irradiation

Let us consider a realistic irradiation of cells with dose-delivery time *T* (h) at dose-rate $\dot{D}$ (Gy/h). In the previous modeling of DNA damage kinetics in a domain during the dose delivery, specific energy (*z*_1_, *z*_2_, …, *z*_N_) is discontinuously deposited in a domain with an amount of DNA (*g*_1_, *g*_2_, …, *g*_N_) at each sub-section of dose-delivery time ([0, *ΔT*), [*ΔT*, 2*ΔT*), …, [(*N *− 1)*ΔT*, *NΔT*))^21^. In addition, the repair rate (*c*_1_, *c*_2_, …, *c*_N_) changes at each sub-section of *T* ([0, *ΔT*), [*ΔT*, 2*ΔT*), …, [(*N *− 1)*ΔT*, *NΔT*))^21^. For the situation, we obtain the relationship of $T = N\Delta T$, where *N* is the number of subsections in total dose-delivery time *T*. As such, the number of PLLs per domain ${x}_{\text{d}}{(}{t}{)}$ can be expressed by

1 $$\begin{gathered} x_{{\text{d}}} (t) = k_{{\text{d}}} g_{1} z_{1} e^{{ - (a{ + }c_{1} )t}} \hfill \{ t | 0 \le t < \Delta T \} \hfill \\ x_{d} (t) = \sum_{n = 1}^{2} k_{{\text{d}}} g_{n} z_{n} e^{{{ - }(a{ + }c_{n} )[t{ - }(n{ - }1)\Delta T]}} \hfill \{ t | \Delta T \le t < 2\Delta T \} \hfill \\ \vdots \hfill \\ x_{{\text{d}}} (t) = \sum_{n = 1}^{N - 1} k_{{\text{d}}} g_{n} z_{n} e^{{{ - }(a{ + }c_{n} )[t{ - }(n{ - }1)\Delta T]}} \hfill \{ t | (N - 2)\Delta T \le t < (N - 1)\Delta T \} \hfill \\ x_{{\text{d}}} (t) = \sum_{n = 1}^{N} k_{d} g_{n} z_{n} e^{{ - (a{ + }c_{n} )[t{ - }(n{ - }1)\Delta T]}} \hfill \{ t | (N - 1)\Delta T \le t \} \hfill \\ \end{gathered}$$

For simplicity, we assume that cell condition (amount of DNA and repair) does not change during irradiation (i.e., *g*_1_ = *g*_2_ = … = *g*_*N*_ = *g* = constant, *c*_1_ = *c*_2_ = … = *c*_*N*_ = *c* = constant). In addition, we consider mean DNA content per domain (i.e., *k*_d_*g* = *k*) for simplicity and the mean dose per nucleus (i.e., < *z*_1_ >  =  < *z*_2_ >  = , …, =  < *z*_*n*_ > = $\dot{z}$ *ΔT*) and take the limit *N* to infinity (hence, *ΔT* → 0). Based on the approximation to express the continuous irradiation^44^, Eq. (1) can be re-expressed as

2 $$\begin{aligned} \mathop {{\text{Lim}}}\limits_{N \to \infty } x_{{\text{d}}} (t) \cong \left\{ {\begin{array}{l} {\mathop \smallint \limits_{0}^{T} \left[ {k\dot{z} - (a + c)x_{\text{d}} (t)} \right]{\text{d}}t \hfill \{ t | t \le T \} } \\ { \mathop \smallint \limits_{0}^{T} \left[ { - (a + c)x_{\text{d}} (t)} \right]{\text{d}}t \hfill \{ t | T < t \} } \\ \end{array} } \right. \hfill \\ = \left\{ {\begin{array}{l} {\frac{{k\dot{z}}}{a + c}[1 - e^{{{\mathfrak{-}}(a + c)t}} ] \hfill \{ t | t \le T \} } \\ { \frac{{k\dot{z}}}{a + c}[1 - e^{{ - (a{ + }c)T}} ] e^{{ - (a{ + }c)(t - T)}} \hfill \{ t | T < t \} } \\ \end{array} } \right. \hfill \\ \end{aligned}$$

where $\dot{z}$ is the specific energy rate (Gy/h). PLL induction competes with the repair during dose delivery, while the LLs gradually increase during and after irradiation. Thus, the number of LLs per domain *w*_d_ can be expressed by

3 $$\begin{aligned} \frac{{\text{d}}}{{{\text{d}}t}}w_{\text{d}} (t) = ax_{\text{d}} (t) + b_{\text{d}} x_{\text{d}} (t)^{2} \hfill \\ w_{{\text{d}}} (t) = \mathop \smallint \limits_{0}^{\infty } \left[ {ax_{\text{d}} (t) + b_{\text{d}} x_{\text{d}} (t)^{2} } \right]{\text{d}}t. \hfill \\ \end{aligned}$$

**Figure 2:**
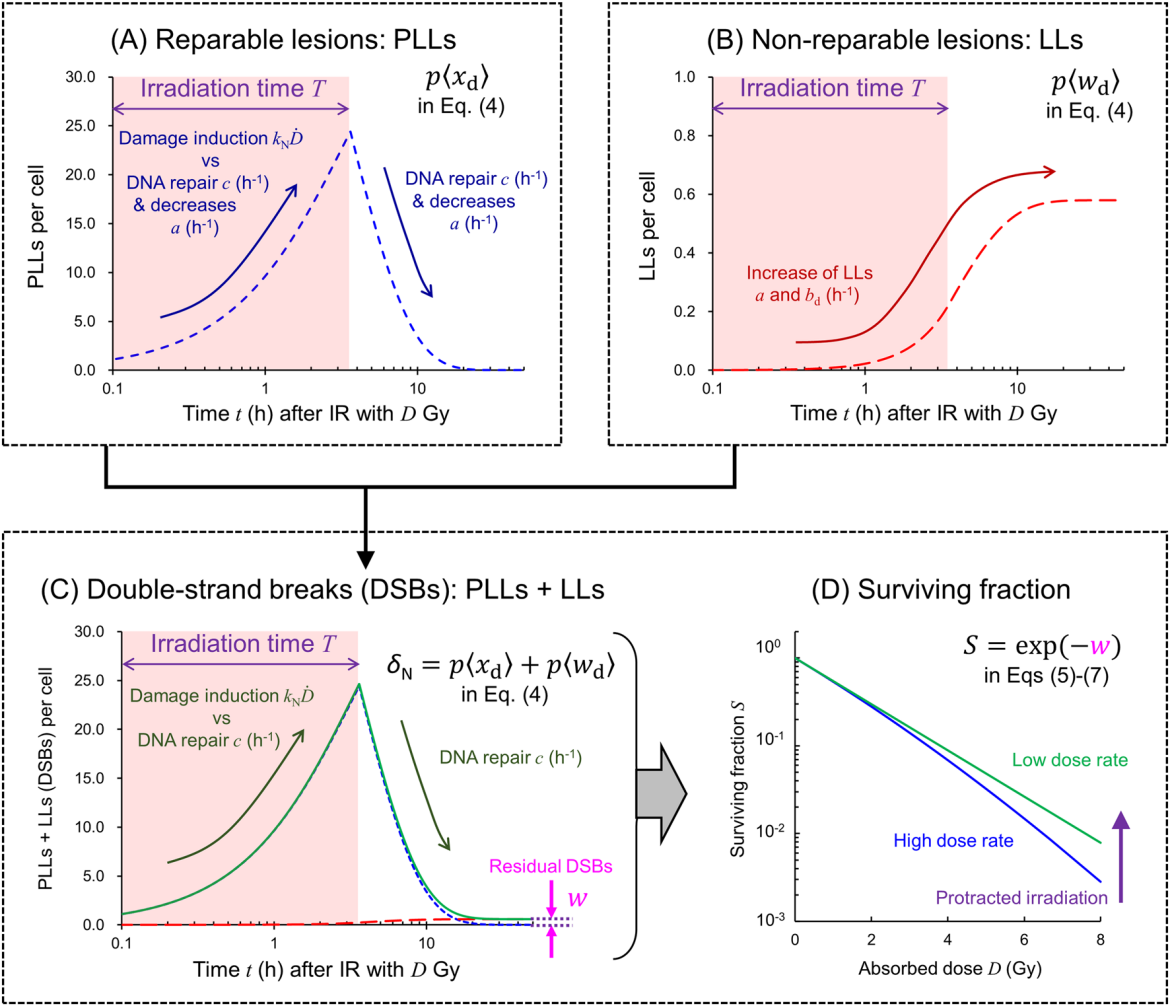
Schematic illustration of the IMK model concept. (**A**) Kinetics of reparable lesion of PLLs, (**B**) that of non-reparable lesion of LLs, (**C**) that of DSBs (the sum of PLLs and LLs), and (**D**) cell surviving fraction. The kinetics of PLLs and LLs in cell nucleus (*p* <*x*_d_> and *p* <*w*_d_>) can be depicted based on Eq. (4). Note that the PLLs and LLs in domains (*x*_d_ and *w*_d_) are expressed by Eqs. (1)–(3). The sum of PLLs and LLs (*δ*_N_) corresponds to the DSBs as depicted in 2C. Assuming Poisson statistics for the number of DSBs remained in cell nucleus (*w*), the surviving fraction is expressed by Eqs. (5)–(7). Using (*a* + *c*) value, cell recovery can be estimated, as depicted in 2D. *DSB* double-strand break, *IMK* integrated microdosimetric, *IR* ionizing irradiation, *PLL* potentially lethal lesion, *LL* lethal lesion.

Considering the mean numbers of PLLs and LLs per domain, < *x*_d_(*t*) > and < *w*_d_(*t*) > , respectively, the sum of PLLs and LLs per cell nucleus, *δ*_N_(*t*), can be given as

4 $$\begin{aligned} {}& \delta_{{\text{N}}} (t) = p\left\langle {x_{{\text{d}}} (t)} \right\rangle + p\left\langle {w_{{\text{d}}} (t)} \right\rangle \hfill = \left\{ {\begin{array}{ll} {\frac{{k_{{\text{N}}} \dot{D}}}{a + c}[1 - e^{ - (a + c)t} ] + \int_{0}^{\infty } {\left[ {a\left\{ {\frac{{k_{{\text{N}}} \dot{D}}}{a + c}[1 - e^{ - (a + c)t} ]} \right\} + bk_{{\text{N}}}^{2} (\gamma \dot{D} + \dot{D}^{2} )\left\{ {\frac{1}{a + c}[1 - e^{ - (a + c)t} ]} \right\}^{2} } \right]{\text{d}}t} } & {\left\{ {t|t \le T} \right\}} \\ {\frac{{k_{{\text{N}}} \dot{D}}}{a + c}[e^{ - (a + c)(t - T)} - e^{ - (a + c)t} ]  + \int_{0}^{\infty } {\left[ {a\left\{ {\frac{{k_{{\text{N}}} \dot{D}}}{a + c}[e^{ - (a + c)(t - T)} - e^{ - (a + c)t} ]} \right\} + bk_{{\text{N}}}^{2} (\gamma \dot{D} + \dot{D}^{2} )\left\{ {\frac{1}{a + c}[e^{ - (a + c)(t + T)} - e^{ - (a + c)t} ]} \right\}^{2} } \right]{\text{d}}t} } & {\left\{ {t|T < t} \right\}} \\ \end{array} } \right. \end{aligned}$$

where *b* = *b*_d_/*p*, *p* is the mean number of domains packaged in a cell nucleus, ${k}_{{\text{N}}} {= pk}$, $\left\langle {\dot{z}^{2} } \right\rangle = \left\langle {\dot{z}} \right\rangle^{2} + \gamma \left\langle {\dot{z}} \right\rangle = \dot{D}^{2} + \gamma \dot{D}$, *γ* = *y*_D_/ρπ*r*_d_^2^, *y*_D_ is the dose-mean lineal energy (keV/μm), ρ and *r*_d_ are the density and the radius of domains, respectively. In this study, we assumed equivalence of the domain to liquid water because cells are mainly composed of water (i.e., at density of 1.0 g/cm^3^), and the radius of 0.5 μm based on the previous study for LET dependence^40^. In Eq. (4), from the previous model study^36,44^, the initial number of PLLs per nucleus and that of LLs at 24 h after irradiation agreed well with the yield of initial DSBs and that of residual DSBs. Figure 2 shows the schematic illustration of the IMK model, where the kinetics of PLLs (panel A) and LLs (panel B) in cell nucleus (*p* < *x*_d_(*t*) > and *p* < *w*_d_(*t*) >) are depicted based on Eqs. (4). Note that the PLLs and LLs in domains (*x*_d_ and *w*_d_) are expressed by Eqs. (1)–(3). The sum of PLLs and LLs (*δ*_N_(*t*)) corresponds to the DSBs as depicted in Fig. 2C. *δ*_N_(*t*) in Eq. (4) therefore describes the DSB kinetics which can be evaluated with the γ-H2AX focus formation assay. Note that the *k*_N_ is the yield of nuclear PLLs (i.e., nuclear DSBs) immediately after irradiation and the (*a* + *c*) can be approximated as *c* which can be obtained from the repair rate of radiation-induced DSBs.

### Surviving fraction as a function of absorbed dose

The IMK model follows the prediction of DSB kinetics during and after irradiation. Using the kinetic equations of PLLs and LLs [i.e., Eqs. (1) and (3)], the surviving fraction can be expressed by the IMK model. Based on the previous modeling^21^, the mean number of LLs in a nucleus *w* and surviving fraction *S* as a function of absorbed dose *D* delivered to cell population during irradiation time (${D} \, \text{=} \, \dot{ \, {D}}{T}$) can be expressed by

5 $$\begin{gathered} - \ln S = w = \left( {\alpha_{0} + \gamma \beta_{0} } \right)\dot{D}T + \frac{{2\beta_{0} }}{{(a{ + }c)^{2} T^{2} }}\left[ {(a{ + }c)T{ + }e^{ - (a + c)T} - 1} \right]\left( {\dot{D}T} \right)^{2} \hfill \\ = \left( {\alpha_{0} + \gamma \beta_{0} } \right)D + F\beta_{0} D^{2} , \hfill \\ \end{gathered}$$

where *α*_0_ and *β*_0_ are the cell-specific coefficients for dose (Gy) and dose squared (Gy^2^), respectively, that are expressed as below,

6a $${\upalpha }_{{0}} { = }\frac{{{ak}_{{\text{N}}} }}{{(a + c)}}{ } \cong { }\frac{{{ak}_{{\text{N}}} }}{{c}},$$

6b $$\beta_{0} = \frac{{bk_{\text{N}}^{2} }}{2(a + c)} \cong \frac{{bk_{\text{N}}^{2} }}{2c},$$

7 $$F = \frac{2}{{(a + c)^{2} T^{2} }}\left[ {(a + c)T + e^{{{ - }(a + c)T}} - 1} \right].$$

*F* corresponds to the Lea-Catcheside time factor^45^. From the comparison between Eq. (7) and the Lea-Catcheside time factor^45^, the present model can describe the sublethal damage repair (SLDR) traditionally defined in radiation biology^22,23^ using the *F* value including the cell-specific parameter (*a* + *c*) (that can be approximated by *c*^46^). The cell-specific parameters (*α*_0_, *β*_0_) can be determined by fitting the Eq. (7) to experimental dose–response curve of the surviving fraction. As depicted in Fig. 2D, the surviving fraction increases as irradiation is protracted. Using Eq. (7), we analyzed the experimental survival data in HLECs and WI-38 cells and quantitatively evaluated the impact of the repair during irradiation at various dose rates (0.033–1.82 Gy/min).

### Determination of the model parameters

To predict the time-dependent nuclear DSBs and cell survival, sets of the model parameters (*k*_N_, *a*, *b, a* + *c*, *γ*) for the DSB kinetics and (*α*_0_, *β*_0_, *γ*, *a* + *c*) for the cell survival need to be determined. Even under assumptions for simplicity, the equations [Eqs. (4) and (5)] are complex and it is difficult to directly determine the model parameters by fitting approach. Thus, we took the following four steps.

(i) The *γ* value of 150 kVp X-rays used in the experiments was obtained using the Monte Carlo track-structure simulations (PHITS^29^ and WLTrack^47^) as described^36^.
(ii) The repair rate *c* (h^−1^) and the initial DSB yield *k*_N_ were obtained by fitting the exponential function (i.e., *k*_N_*e*^*−ct*^) to the measured DSB repair kinetics after acute irradiation (1 Gy at 1.82 Gy/min) with the least square method. The *c* value was used as prior information of (*a* + *c*).
(iii) Using the *γ* and (*a* + *c*) values, the cell-specific coefficients (*α*_0_, *β*_0_) were determined by fitting Eq. (7) to the experimental dose–response curve of surviving fraction after acute irradiation with the Markov chain Monte Carlo (MCMC) simulation. Note that the (*a* + *c*) value was updated due to the cell-specific parameters in the MCMC simulation.
(iv) Last, the cell*-*specific parameters for DSB estimation (*a*, *b*) were obtained with Eq. (6), the *k*_N_ value given by step (i), and the coefficients (*α*_0_, *β*_0_) determined by step (ii).

In this fitting approach, we used the MCMC simulation which allows the estimation of the uncertainties of model parameters, details of which have been described^48^. In this simulation, assuming a uniform distribution for the cell-specific coefficients (*α*_0_, *β*_0_) as the prior distribution. By using the likelihood *P*(*d*_*i*_|*θ*) and the transition probability *α*_P_, the set of cell-specific parameters $\theta$[*α*_0_, *β*_0_, *a* + *c*] was sampled as follows:

8 $$P(d|\theta ) = \mathop \prod \limits_{i = 1}^{N} [P(d_{i} |\theta )] = \mathop \prod \limits_{i = 1}^{N} \left\{ {\frac{1}{{\sqrt {2\pi \sigma } }}\exp \left[ { - \frac{{( - \ln S_{\text{exp}\, i } + \ln S_{\text{cal}\, i } )^{2} }}{{2\sigma^{2} }}} \right]} \right\}$$

9 $$\alpha_{{\text{P}}} = \frac{{P(\theta^{{{\text{candidate}}}} | d)}}{{P(\theta^{(t)} |d)}}$$

where *d*_*i*_ (*i* = 1~*N*) is the experimental data [i.e., *d*_*i*_ = (*D*_*i*_, − ln *S*_exp*i*_)], *S*_exp_ is the measured surviving fraction, *S*_cal_ is the predicted surviving fraction, and *P*(*θ*|*d*) and* P*(*θ*^*candidate*^|*d*) are the posterior likelihood for the candidate (*t* + 1)-th and the previous *t*-th conditions, respectively. After the MCMC simulation, we calculated the mean and standard deviation (sd) of the model parameters of (*α*_0_, *β*_0_, *a* + *c*). The sd values of *a* and *b* were calculated using error propagation formula with the sd values of *k*_N_, *α*_0_, *β*_0_, *a* + *c* that were obtained by the steps of (ii) and (iii). The burn-in and sampling numbers were defined as 10^3^ and 10^4^, respectively.

### Estimation of DSB kinetics and surviving fractions

Using the model parameters determined by using the experimental data after acute irradiation, we estimated the time-dependent nuclear DSB and surviving fraction of HLECs and WI-38 cells. As for the DNA damage analysis, we used the parameter set of (*k*_N_, *a*, *b, a* + *c*, *γ*) and Eq. (4), and estimated the number of nuclear DSBs at various times of X-irradiation. The estimated DSBs were verified by comparison to the number of nuclear γ-H2AX foci (experimental DSBs) at 0.5, 1, 2.5, 6, 24, and 48 h after acute exposure at 1.82 Gy/min. In evaluating dose-rate dependence, the estimated DSBs were also compared to the measured DSBs at 0.5 h after the end of irradiation and 48 h after the start of irradiation at five different dose rates. Using the parameter set of (*α*_0_, *β*_0_, *γ*, *a* + *c*) and Eq. (5), we estimated the surviving fractions in HLECs and WI-38 cells. In addition, we calculated the uncertainties of the estimated values (corresponding to the standard error of 1σ) by using the standard errors of the model parameters. Based on the comparison between the estimation by the model and the experimental cell survival after irradiation with 2 and 4 Gy, we evaluated the impacts of dose rate on the surviving fraction.

### Estimation accuracy

To discuss how simulated estimates differ from experimental data, we used the coefficient of determination *R*^2^ value as statistical measures. The *R*^2^ value is given by

10 $$R^{2} = 1 - \frac{{\sum_{i = 1}^{n} (\exp_{i } - {\text{cal}}_{i } )^{2} }}{{\sum_{i = 1}^{n} (\exp_{i } - \left\langle {\exp } \right\rangle )^{2} }},$$

where exp_*i*_ is measured cell survival, and cal_*i*_ is cell survival calculated by the present model.

## Results and discussions

### DSB kinetics and cell survival after acute irradiation

We first measured the repair kinetics of DSBs and the surviving fraction of HLECs after acute irradiation. The radiation-induced DSBs were measured by a γ-H2AX focus formation assay, while the surviving fraction was obtained using a clonogenic assay. In addition, we applied the IMK model [i.e., Eqs. (4) and (5)] to the experimental data of DSBs and cell survival after acute irradiation to obtain the cell-specific model parameters.

**Figure 3:**
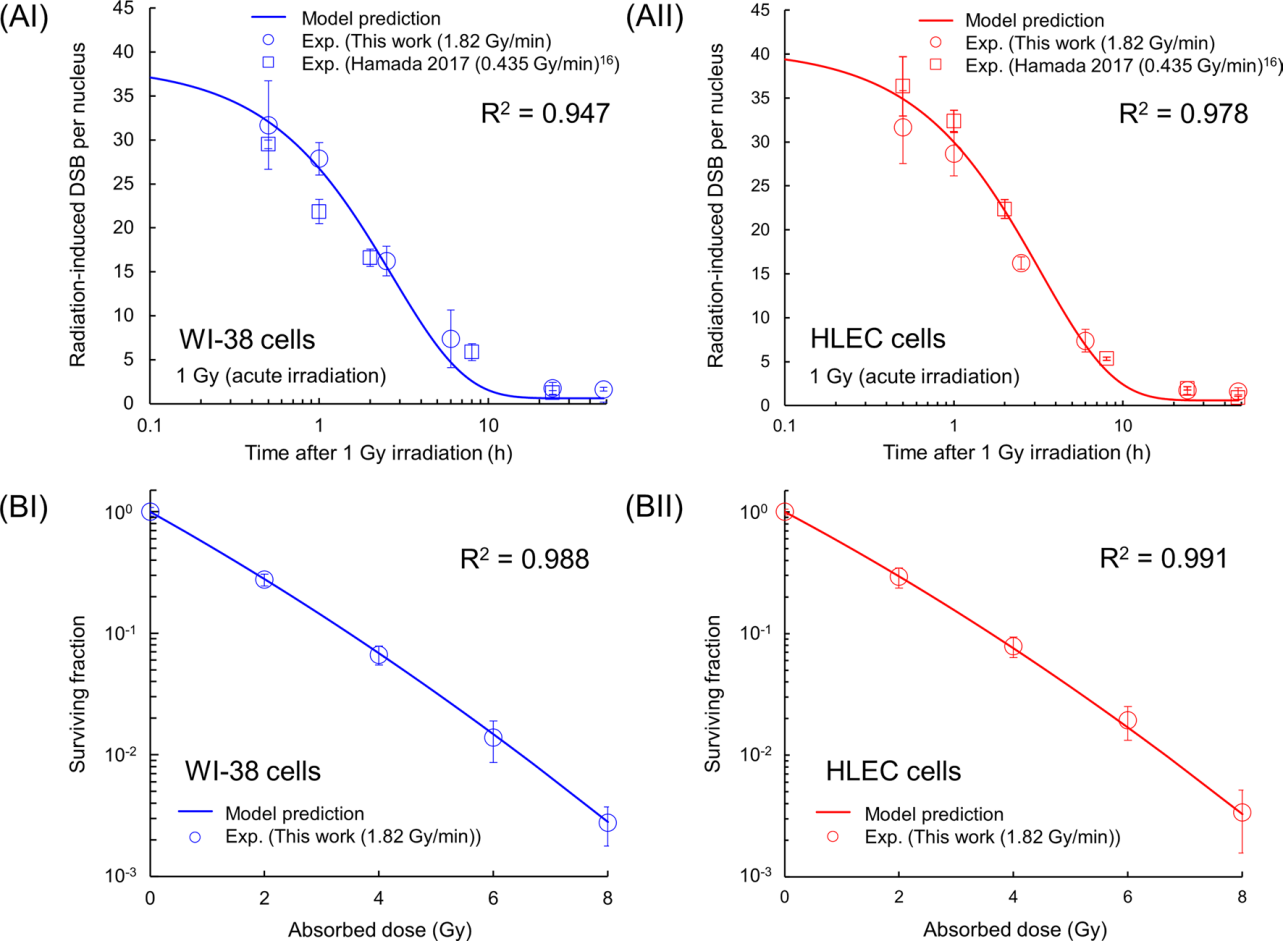
DSB kinetics and cell surviving fraction after acute irradiation. The experimental data of acute irradiation were obtained with 150 kVp X-rays (Hamada^16^ and this work). The dose rates in Hamada 2017 and this work were 0.435 and 1.82 Gy/min, respectively. (**A**) DSB kinetics after acute irradiation of WI-38 cells (I) and HLECs (II). (**B**) Cell survival of WI-38 cells (I) and HLECs (II). The symbols and the solid line represent experimental data and model prediction, respectively.

The repair kinetics of DSBs and the dose–response curve of surviving fraction in WI-38 cells and HLECs were shown in Fig. 3A,B, where symbols (circles and squares) and solid lines represent the experimental data and the estimate by the IMK model [i.e., Eqs. (4) and (5)], respectively. The model parameters used for the estimation are listed in Table 1. Judging from the *R*^2^ value in Fig. 3A,B, the estimated curves agreed well with the experimental results given that the IMK models for DSB [Eq. (4)] and for cell survival [Eq. (5)] were fitted to the corresponding experimental data. The experimental DSB data (the previous experimental data^16^ and the present data) for acute irradiation (i.e., 0.435 and 1.82 Gy/min in Hamada^16^ and this work, respectively) shows that repair of radiation-induced DSB can complete within 24 h after the start of irradiation (Fig. 3AI, II). Against the experimental DSB results, the model predicted the DSB repair rates (*a* + *c* $\cong$ *c* [h^−1^] in the IMK model) of 0.309 $\pm$ 0.056 (h^−1^) in HLECs and 0.371 $\pm$ 0.038 (h^−1^) in WI-38 cells, suggesting slower DSB repair in HLECs than in WI-38 cells. Note that the *a* value is smaller than a few percent of *c* value (see Table 1). Due to a repair rate, the impact of recovery of HLECs during irradiation can be considered small, which agrees well with the ICRP assumption of no dose rate effects^10^. On the other hand, the surviving fraction of HLECs was similar to that of WI-38 cells consistent with the previous report^15^, confirming that cell killing may not underlie the high radiosensitivity of the lens. Meanwhile, we used HLECs derived from a 20-week gestation male as a donor, and did not address the potential inter-individual difference in radiation responses of HLECs derived from various donors. In the future study, it is of great importance to clarify the DNA repair mechanisms in HLECs (e.g., derived from cells of patients with degenerative and inflammatory syndromes).

**Table 1:** IMK model parameters for WI-38 cells and HLECs.

Model parameters	Cell lines						Note
	WI-38 cells			HLECs			
*k*_N_ (Gy^−1^)	3.84 × 10^1^	±	1.70 × 10^0^	4.06 × 10^1^	±	2.63 × 10^0^	*δ*_N_ prediction [Eq. (4)]
*a* (h^−1^)	5.59 × 10^–3^	±	1.20 × 10^–3^	4.27 × 10^–3^	±	1.07 × 10^–3^	*δ*_N_ prediction [Eq. (4)]
*b* (h^−1^)	8.17 × 10^–6^	±	6.73 × 10^–6^	6.58 × 10^–6^	±	4.90 × 10^–6^	*δ*_N_ prediction [Eq. (4)]
*a* + *c* (h^−1^)	3.71 × 10^–1^	±	3.84 × 10^–2^	3.09 × 10^–1^	±	5.58 × 10^–2^	*δ*_N_ & *S* prediction [Eq. (4) and (5)]
*α*_0_ (Gy^−1^)	5.90 × 10^–1^	±	1.07 × 10^–1^	5.59 × 10^–1^	±	9.51 × 10^–2^	*S* prediction [Eq. (5)]
*β*_0_ (Gy^−2^)	1.63 × 10^–2^	±	1.32 × 10^–2^	1.75 × 10^–2^	±	1.24 × 10^–2^	*S* prediction [Eq. (5)]
γ (Gy)	9.54 × 10^–1^			9.54 × 10^–1^			*δ*_N_ and *S* prediction [Eqs. (4) and (5)]

The model parameters were determined by fitting the model to the experimental DSB and cell survival data after acute exposure. Note that the model parameter sets (*k*_N_, *a*, *b, a* + *c*,* γ*) and (*α*_0_, *β*_0_, *γ*, *a* + *c*) are for the DSB kinetic and cell survival estimations, respectively.

### Experimental time-dependent nuclear DSB for various dose rates

We next measured the time-dependent DSBs after exposure at various dose rates, i.e., 1.82 Gy/min, 0.1 Gy/min, 0.033 Gy/min, 0.00461 Gy/min (27.7 cGy/h), and 0.00081 Gy/min (4.9 cGy/h). Both WI-38 cells and HLECs were fixed at 0.5 and 1 h after the end of irradiation and 24 and 48 h after the start of irradiation. From the experimental data on nuclear γ-H2AX foci, we evaluated the impact of DSB repair during and after exposure and dose-rate dependence.

**Figure 4:**
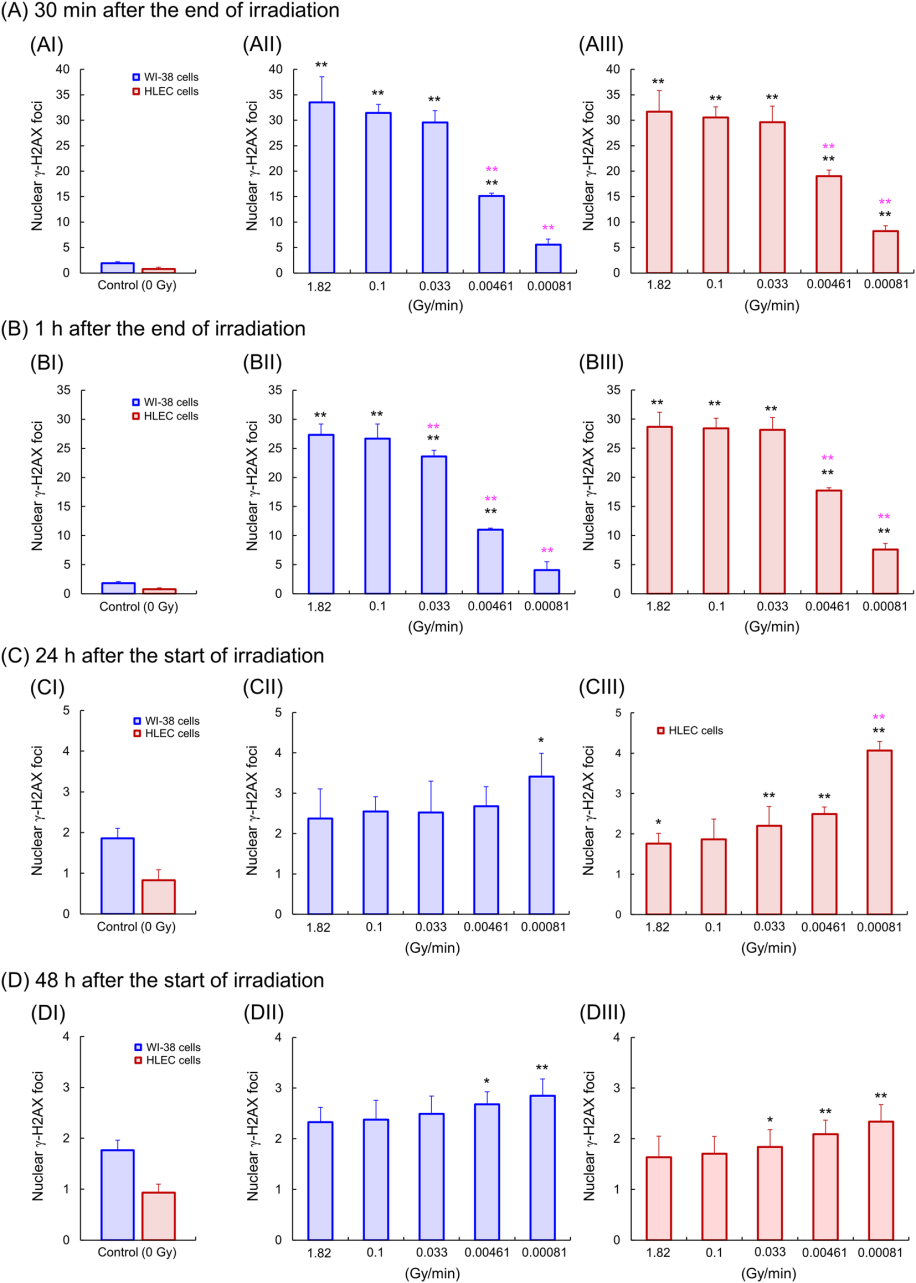
Time-dependent nuclear γ-H2AX foci. (**A**) 30 min after the end of irradiation, (**B**) 1 h after the end of irradiation, (**C**) 24 h after the start of irradiation, and (**D**) 48 h after the start of irradiation. The blue and red histograms represent WI-38 cells and HLECs, respectively. The black asterisks * and ** indicate 5% and 1% significant difference between a control group and an irradiated one. The colored asterisks indicate * and ** are 5% and 1% significant difference between a high dose rate irradiated group (1.82 Gy/min) and a certain irradiated group.

Figure 3 depicts the time-dependent nuclear γ-H2AX foci for five different dose rates, where (A) is nuclear γ-H2AX foci at 30 min after the end of irradiation, (B) 1 h after the end of irradiation, (C) 24 h after the start of irradiation, and (D) 48 h after the start of irradiation. Figures S2 and S3 show the distribution of nuclear γ-H2AX foci in WI-38 cells and HLECs at 30 min and 1 h after the end of irradiation and 24 h and 48 h after the start of irradiation. As seen in Fig. 4AII, III as well as Figs. S2 and S3, the significant repair effects during the dose-delivery (irradiation) time at 0.00461 Gy/min (27.7 cGy/h) and 0.00081 Gy/min (4.9 cGy/h) for both cell types (see the colored ** in Fig. 4AII, III). Focusing on 30 min after the end of irradiation at 0.00081 Gy/min (4.9 cGy/h), the remaining γ-H2AX foci during irradiation only in the HLECs (significant γ-H2AX foci induction after the irradiation) were observed, indicating the slower repair rate in HLECs compared to WI-38 cells (see the black ** in Fig. 4AII, III). Reflecting the different repair rates, the nuclear γ-H2AX foci of WI-38 cells 1 h after the end of irradiation at 0.033 Gy/min were significantly reduced by virtue of the higher repair rate compared to those of HLECs (see the colored ** in Fig. 4BII, III). This trend can be confirmed by the nuclear γ-H2AX foci distribution shown in Figs. S2 and S3. The nuclear foci in WI-38 cells 30 min and 1 h after the end of irradiation at 0.03 Gy/min were also significantly reduced compared to those at 0.1 Gy/min, whereas there was no significant repair in HLECs (see Tables S1–S8). These trends also show the slower repair rate in HLECs compared to WI-38 cells. At 24 h after the start of irradiation, the nuclear γ-H2AX foci in WI-38 cells were completely repaired at 0.00461–1.82 Gy/min, while those in HLECs remained without completely repaired (see Fig. 4CII, III). As for the γ-H2AX foci at the lowest dose rate (i.e., 0.00081 Gy/min (4.9 cGy/h)), significant γ-H2AX foci remained in both cell lines. This is because the period after the end of irradiation is short (ca. 3.4 h). At 48 h after the start of irradiation corresponding to a sufficiently long period after the end of irradiation, the residual γ-H2AX foci for both cell lines showed the tendency of IDREs such that the γ-H2AX foci increase as dose rate decreases (see Fig. 4DII, III). This result is consistent with the trend in Barnard et al.^18^ Particularly, γ-H2AX foci remained only in HLECs irradiated at 0.033 Gy/min, indicating the IDRE is more significant in HLECs than in WI-38 cells.

In this study, using highly fractionated irradiation, we generated the intermediate dose rates, i.e., 0.033 and 0.1 Gy/min. Strictly speaking, multi-fractionation and continuous irradiation may vary each other from the viewpoints of competition between DNA damage repair production during dose delivery. Compared to continuous irradiation, irradiation with multiple pulses can cause more DNA damage instantaneously, which can potentially induce more residual DNA damage (corresponding to LLs defined in the IMK model). Meanwhile, in our previous studies^18,45^, theoretical predictions using the IMK model have confirmed that if multiple small doses are delivered to cells by hyper-fractionation (i.e., 6.0 Gy/h: 2.0 Gy/Fr at 20 min interval, 3.0 Gy/h: 1.5 Gy/Fr at 30 min interval, 1.0 Gy/h: 1 Gy/Fr at 1 h interval, and 1.0 Gy/h: 0.186 Gy/Fr at 1 h interval)^21,49^, these irradiations are likely similar to each other, leading to similar biological responses as those of continuous irradiation. Considering these, highly fractionated irradiation is an effective method to observe the biological effects after exposure at intermediate dose rates of 0.033 and 0.1 Gy/min.

### Model analysis for DNA damage responses

**Figure 5:**
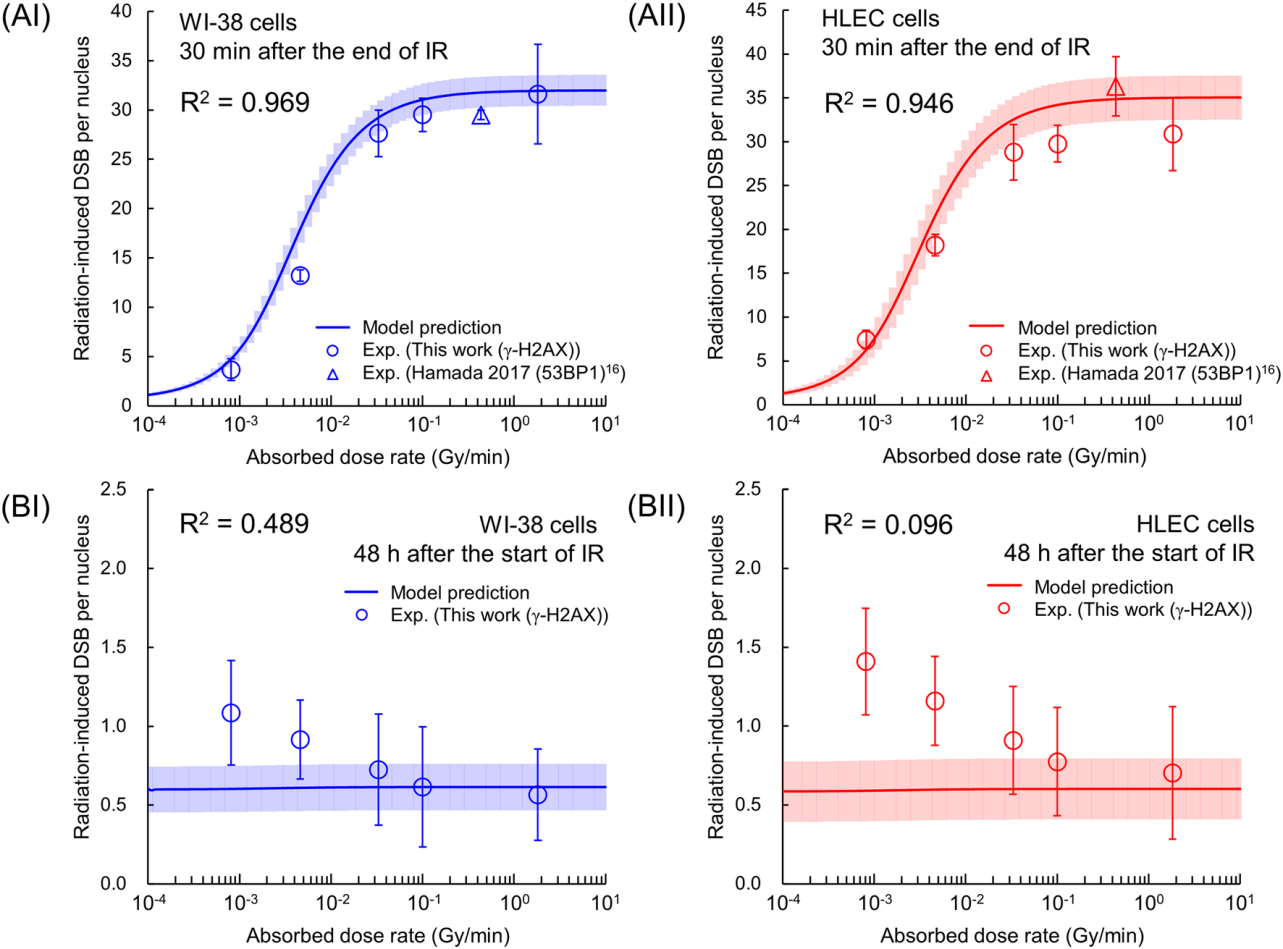
Nuclear DSBs as a function of dose rate. (**A**) 30 min after the end of irradiation, and (**B**) 48 h after the start of irradiation. The symbols and the solid line represent experimental data and model prediction, respectively. As seen in panels BI and BII, the inverse-dose-rate effects were observed compared to those at a highest dose rate, i.e., 1.82 Gy/min. The colored area represents the standard error (corresponding to 1σ) of the estimated value.

To further discuss the dose-rate dependence, we estimated the number of nuclear DSBs at 30 min after the end of irradiation and 48 h after the start of irradiation, as shown in Fig. 5A,B. The model estimation for 30 min after the end of irradiation shows that the impacts of repair for both WI-38 cells and HLECs during irradiation become significant in the dose-rate range of < 0.033 Gy/min, which agreed well with the experimental data based on the *R*^2^ value in Fig. 5A. In the supplementary file, Fig. S4 compares the model estimations and the experimental data of the nuclear DSBs as a function of time after the start of irradiation, i.e., 0 − 48 h, at 0.00081 − 1.82 Gy/min, where the IMK model successfully reproduced the tendency of the experimental data. From the comparison in Fig. S4 and the experimental data in Figs. S2 and S3, the slower repair rate of HLECs was verified. The Bodgi function^51^ has been proposed as a mathematical model that allows the precise estimation of phosphorylation processes within 30 min after irradiation and the repair kinetics. However, the function does not consider the competition between DSB induction and its repair processes during protracted irradiation which have been incorporated into the IMK model (i.e., our model). In future, by combing the Bodgi function^51^ and the IMK model, it might be possible to better reproduce the experimental, time-dependent γ-H2AX foci. Meanwhile, for 48 h after the start of irradiation, the model estimation showed a slight reduction of residual γ-H2AX foci (Fig. 5B). However, there was a discrepancy between the mean estimation values and the corresponding experimental residual DSBs (Fig. 5BI, II). The model analysis suggested that the measured residual γ-H2AX foci at 0.00081 Gy/min (4.9 cGy/h) is approximately 1.8 and 2.5 times as much as the model predictions for WI-38 cells and HLECs, respectively. Considering the experimental DSBs and the model predictions, the IDREs are of importance in evaluating radiation effects on the lens. Meanwhile, there are several other markers for detecting early DSB induction after irradiation, such as ataxia telangiectasia mutated (ATM) and 53BP1^50^. Further investigation of IDREs to look at co-localization of such foci is necessary in the future study.

One potential cause of IDREs for DNA damage may be radiation-induced bystander effects (RIBEs) (so-called non-targeted effects)^52–55^. The RIBEs are induced by intercellular signaling under heterogenous exposure^56,57^. During protracted exposure at a low dose rate, energy deposition is expected to be spatiotemporally heterogeneous and may result in enhanced radiosensitivity due to the stacking of the RIBEs. Meanwhile, there is no significant increase of RIBEs-induced biological effects in the out-of-field cells by a split-dose experiment with modulated radiation field exposure (i.e., 50% in-field area and 50% out-of-field area)^58,59^. However, because there is limited scientific data on DNA damage responses, the involvement of RIBEs during protracted exposure remains unclear. Further exploration of the relation of DNA damage responses and RIBE at low dose rate is mandatory. The other potential cause of IDREs is the change in cell cycle phases during exposure^21,60^. The radiosensitivity for cell survival depends on the cell cycle phase^61^, as well as the DNA damage response^33^. The accumulation of cells in highly sensitive phases such as G_2_ and M during protracted exposure might lead to IDREs^21,62,63^. Particularly, HLECs show excessive proliferation, which may be associated with cell-cycle-dependent radiosensitivity (cell-cycle-based IDREs). Considering the excess proliferation of HLECs, it is more likely that a cell cycle phase transition during low-dose-rate exposure resulted in higher levels of γ-H2AX foci^33^. However, HLECs grow slowly (i.e., doubling time: 41.6 $\pm$ 8.8 h) (see Fig. S5), suggesting small contribution of cell-cycle change to IDRE induction. Although there are potential causes leading to IDREs, the underlying mechanisms behind IDREs in HLECs remain unclear. Further biological studies are needed to understand dose-rate effects on the lens. In addition, from the viewpoint of radiation quality, exposures at higher dose rates (i.e., 1.82, 0.1, and 0.033 Gy/min) were performed by acute or fractionated dose delivery with 150 kVp X-rays whereas those at lower dose rates (i.e., 0.00461 Gy/min (27.7 cGy/h) and 0.00081 Gy/min (4.9 cGy/h)) were done by continuous dose delivery with ^137^Cs γ-rays. Our previous DNA damage simulation using an electron track-structure simulation of PHITS revealed the DSB yield (/Da/Gy) for ^137^Cs γ-rays is lower than that for 150 kVp X-rays^64^. The IDREs for residual DSBs become more significant than the results shown in Fig. 4, thereby indicating that the discrepancy between the model prediction and the experimental residual DSBs can be increased. The yields of DSBs estimated by the PHITS code^29^ are summarized in Fig. S6, in which the ratio of DSB yield for ^137^Cs γ-rays to that for 150 kVp X-rays is 0.92.

### Cell survival for various dose rates

We finally investigated the surviving fraction after exposure at various dose rates, i.e., 1.82, 0.435, 0.1, and 0.033 Gy/min, using a clonogenic assay. Note that the experimental survival data at 0.435 Gy/min was obtained from the previous report by Fujimichi and Hamada^15^. Against the measured survival data after irradiation with 2 or 4 Gy, we estimated the surviving fraction as a function of dose rate by using the IMK model [Eq. (5)] and evaluated the dose-rate dependence.

**Figure 6:**
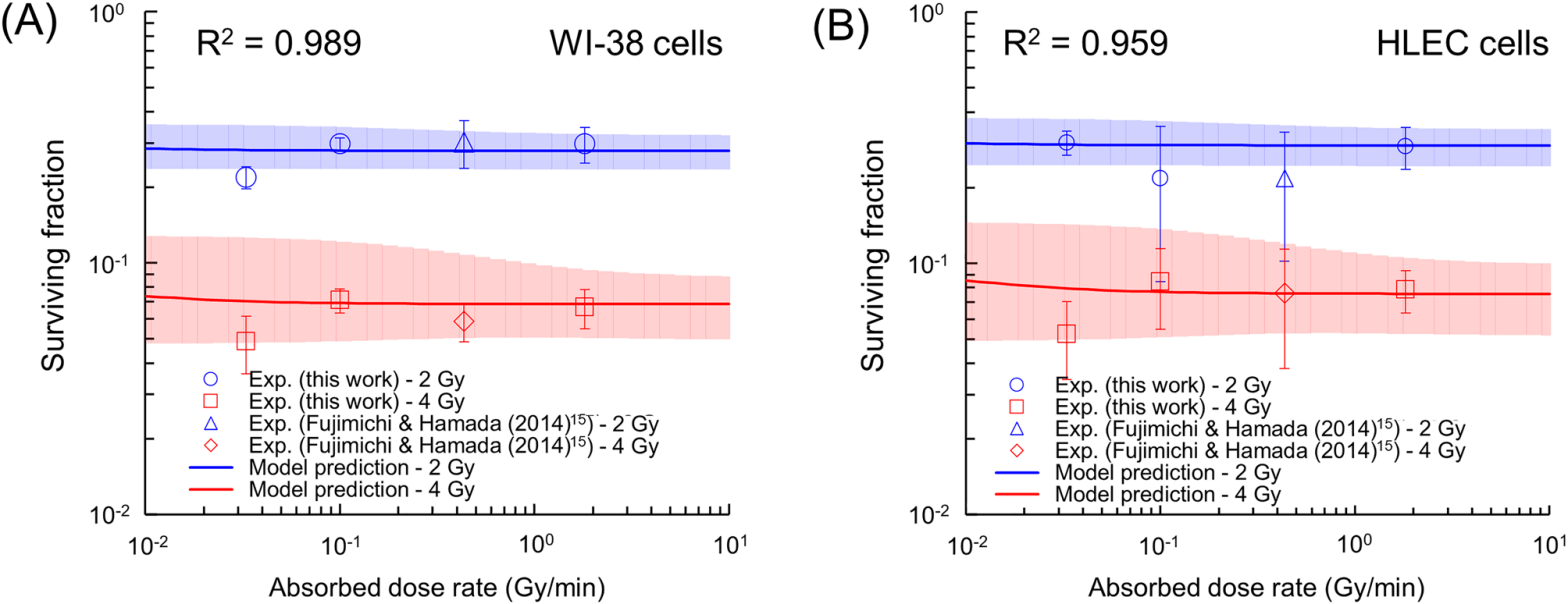
Cell survival as a function of dose rate. (**A**) WI-38 cells, and (**B**) HLECs. The symbols (circles and triangles) and the solid line represent experimental data (2 Gy and 4 Gy) and the model prediction, respectively. Note that the experimental survival data at 0.435 Gy/min was taken from Fujimichi and Hamada^15^. There was no significant dose-rate effect in the experiments, but cell survival at 0.033 Gy/min and 4 Gy tended to be lower compared to that at the highest dose rate (i.e., 1.82 Gy/min) indicated as the dotted black line. The colored area represents the standard error (corresponding to 1σ) of the estimated value.

The comparisons between the experimental survival and the model predictions for WI-38 cells and HLECs are shown in Fig. 6A,B: the experimental results showed no significant difference, but the model prediction exhibited less dose-rate dependence. The dose–response curves of both cell lines for 1.82, 0.453, 0.1, and 0.033 Gy/min are depicted in Fig. S7 of the supplementary file, where the predicted curves overlapped with that at a highest dose rate, i.e., 1.82 Gy/min. From Fig. 6, despite no significant differences, the surviving fraction after exposure at 0.033 Gy/min tended to be lower than at 0.1–1.82 Gy/min. The experimental results for DSBs showed the IDRE below 0.033 Gy/min. This trend of cell survival might be because of the stacking of RIBEs or hyper-radiosensitivity (HRS)^65^. Low-dose fractionated exposures have been reported to enhance the raiosensitivity^66,67^, so this tendency obtained by fractionated irradiation is consistent with the literature^66,67^. Due to the limitation of experimental settings, it is difficult to measure the surviving fraction at 0.00461 Gy/min (27.7 cGy/h) and 0.00081 Gy/min (4.9 cGy/h) in this study. However, the comparison between the model prediction and the experimental survival suggests that there is no significant dose-rate dependence in the dose-rate range of 0.033–1.82 Gy/min. Due to the insufficient evidence of dose rate effects on cataracts, ICRP provided no firm conclusions to date^7^. Thus, ICRP assumes that the threshold is 0.5 Gy independent of dose rate, in other words, no dose rate effects^10^. The trend (Fig. 5) supports the ICRP assumption of no dose rate effects. However, the present data was obtained in vitro, therefore the accumulation of in vivo evidence for cataracts is needed to further characterize the dose rate effects on the lens.

## Conclusion

This work investigated time-dependent nuclear DSBs and cell survival in HLECs after exposure to photons at various dose rates (0.00081–1.82 Gy/min). The combined approach with experiments and simulation revealed that the repair rate is lower in HLECs than in WI-38 cells. As for cell survival there was no significant impact of dose rate in both cell lines at 0.033–1.82 Gy/min, supporting the ICRP assumption of no dose rate effects. On the other hand, the experimental residual DSBs showed IDREs, which exhibited the discrepancy with the prediction by the IMK model. This highlights the importance of IDREs in evaluating radiation effects on the lens.

The long-term goal of this project is to develop a biophysical model for predicting cataractogenesis considering dose-rate effects. To date, the existing model for reproducing cataractogenesis allows the prediction of a relationship between dose and cataract onset at various ages^24^. Ideally, replacing the DNA repair obtained in this study would make the model more accurate. However, as observed in this study, IDRE is a crucial issue to express the dose-rate effects on the lens, warranting further studies in vitro, in vivo, and in silico.

### Supplementary Information

Supplementary Information.

## Data Availability

All data generated or analysed during this study are included in this published article and its supplementary information files.
