# Supplementary material for: The impact of dose rate on responses of human lens epithelial cells to ionizing irradiation
Source: Sci Rep. 2024 May 28;14:12160. doi: 10.1038/s41598-024-62679-8 (PMC11130169; doi:10.1038/s41598-024-62679-8)
Supplement: Supplementary file 1 — Supplementary Information. [file 41598_2024_62679_MOESM1_ESM.pdf]

Supplemental experimental data and model analysis for cellular responses of human lens epithelial cells after photon irradiations

Supplementary Material of "The impact of dose rate on responses of human lens epithelial cells to ionizing irradiation"

This supplementary file presents the cell-cycle distribution of two normal human cell lines, the additional analysis of nuclear DSBs, cell surviving fraction estimated by the integrated microdosimetric-kinetic (IMK) model[1], and the initial DNA damage estimation by PHITS[2]. This file includes 6 figures, Figure S1: cell-cycle distribution of human lung fibroblast cells WI-38 and human lens epithelial cells HLEC, Figure S2: distribution of nuclear γ-H2AX foci in WI-38, Figure S3: distribution of nuclear γ-H2AX foci in HLEC, Figure S4: comparison of time-dependent DSBs between the experiments and the model prediction, Figure S5: Growth curve and doubling time, Figure S6: DNA damage yields estimated by the PHITS code, Figure S7: Dose-response curve of cell survival at various dose rates, Table S1: statistical results for dose-rate dependence of nuclear DSB in WI-38 for 30 min after the end of irradiation, Table S2: statistical results for dose-rate dependence of nuclear DSB in WI-38 for 1 h after the end of irradiation, Table S3: statistical results for dose-rate dependence of nuclear DSB in WI-38 for 24 h after the start of irradiation, Table S4: statistical results for dose-rate dependence of nuclear DSB in WI-38 for 48 h after the start of irradiation, Table S5: statistical results for dose-rate dependence of nuclear DSB in HLEC for 30 min after the end of irradiation, Table S6: statistical results for dose-rate dependence of nuclear DSB in HLEC for 1 h after the end of irradiation, Table S7: statistical results for dose-rate dependence of nuclear DSB in HLEC for 24 h after the start of irradiation, Table S8: statistical results for dose-rate dependence of nuclear DSB in HLEC for 48 h after the start of irradiation.

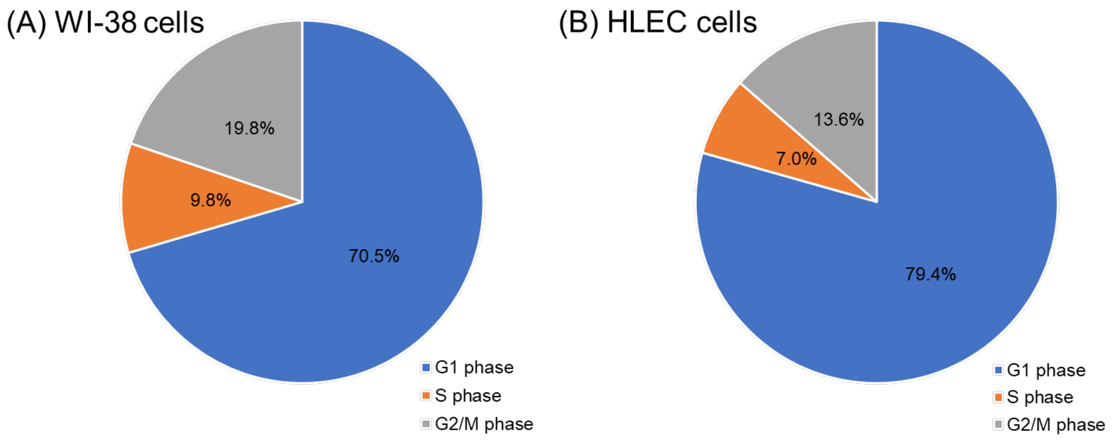

Figure S1. Cell-cycle distribution of human lung fibroblast cells WI-38 and human lens epithelial cells HLEC. (A) WI-38 cells, (B) HLEC. To obtain the fractions of G1, S, G2/S phases, we performed flow-cytometric analysis with PI staining solution. As the method, first, less than 1×10^6 cells were fixed with 70% ethanol (in 0.5 mL of PBS and 4.5 mL of 70% ethanol) and kept at 4°C for at least 2 h. After centrifugation, the cells were suspended in 1 mL PBS. After centrifugation for 10 min at 300g at 4°C, ethanol was thoroughly removed (or decanted). Cells were then treated with 0.5 mL of FxCycle^{TM} PI/RNase staining solution (Life Technologies) with 0.2% v/v tritonX and kept in the dark at 37°C for 15 min.

	1.82 Gy/min	0.1 Gy/min	0.033 Gy/min	0.00461 Gy/min	0.00081 Gy/min
Control (0 Gy)	**	**	**	**	ns
1.82 Gy/min		ns	ns	**	**
0.1 Gy/min			**	**	**
0.033 Gy/min				**	**
0.00461 Gy/min					**
0.00081 Gy/min					

Table S1. Statistical results for dose-rate dependence of nuclear DSB in WI-38 for 30 min after the end of irradiation. * and ** are 5% and 1% significant difference.

	1.82 Gy/min	0.1 Gy/min	0.033 Gy/min	0.00461 Gy/min	0.00081 Gy/min
Control (0 Gy)	**	**	**	**	ns
1.82 Gy/min		ns	ns	**	**
0.1 Gy/min			*	**	**
0.033 Gy/min				**	**
0.00461 Gy/min					**
0.00081 Gy/min					

Table S2. Statistical results for dose-rate dependence of nuclear DSB in WI-38 for 1 h after the end of irradiation. * and ** are 5% and 1% significant difference.

	1.82 Gy/min	0.1 Gy/min	0.033 Gy/min	0.00461 Gy/min	0.00081 Gy/min
Control (0 Gy)	ns	ns	ns	ns	*
1.82 Gy/min		ns	ns	ns	ns
0.1 Gy/min			ns	ns	ns
0.033 Gy/min				ns	ns
0.00461 Gy/min					ns
0.00081 Gy/min					

Table S3. Statistical results for dose-rate dependence of nuclear DSB in WI-38 for 24 h after the start of irradiation. * and ** are 5% and 1% significant difference.

	1.82 Gy/min	0.1 Gy/min	0.033 Gy/min	0.00461 Gy/min	0.00081 Gy/min
Control (0 Gy)	ns	ns	ns	*	**
1.82 Gy/min		ns	ns	ns	ns
0.1 Gy/min			ns	ns	ns
0.033 Gy/min				ns	ns
0.00461 Gy/min					ns
0.00081 Gy/min					

Table S4. Statistical results for dose-rate dependence of nuclear DSB in WI-38 for 48 h after the start of irradiation. * and ** are 5% and 1% significant difference.

	1.82 Gy/min	0.1 Gy/min	0.033 Gy/min	0.00461 Gy/min	0.00081 Gy/min
Control (0 Gy)	**	**	**	**	*
1.82 Gy/min		ns	ns	**	**
0.1 Gy/min			ns	**	**
0.033 Gy/min				**	**
0.00461 Gy/min					**
0.00081 Gy/min					

Table S5. Statistical results for dose-rate dependence of nuclear DSB in HLEC for 30 min after the end of irradiation. * and ** are 5% and 1% significant difference.

	1.82 Gy/min	0.1 Gy/min	0.033 Gy/min	0.00461 Gy/min	0.00081 Gy/min
Control (0 Gy)	**	**	**	**	**
1.82 Gy/min		ns	ns	**	**
0.1 Gy/min			ns	**	**
0.033 Gy/min				**	**
0.00461 Gy/min					**
0.00081 Gy/min					

Table S6. Statistical results for dose-rate dependence of nuclear DSB in HLEC for 1 h after the end of irradiation. * and ** are 5% and 1% significant difference.

	1.82 Gy/min	0.1 Gy/min	0.033 Gy/min	0.00461 Gy/min	0.00081 Gy/min
Control (0 Gy)	*	ns	**	**	**
1.82 Gy/min		ns	ns	ns	**
0.1 Gy/min			ns	ns	**
0.033 Gy/min				ns	**
0.00461 Gy/min					**
0.00081 Gy/min					

Table S7. Statistical results for dose-rate dependence of nuclear DSB in HLEC for 24 h after the start of irradiation. * and ** are 5% and 1% significant difference.

	1.82 Gy/min	0.1 Gy/min	0.033 Gy/min	0.00461 Gy/min	0.00081 Gy/min
Control (0 Gy)	ns	ns	*	**	**
1.82 Gy/min		ns	ns	ns	ns
0.1 Gy/min			ns	ns	ns
0.033 Gy/min				ns	ns
0.00461 Gy/min					ns
0.00081 Gy/min					

Table S8. Statistical results for dose-rate dependence of nuclear DSB in HLEC for 1 h after the end of irradiation. * and ** are 5% and 1% significant difference.

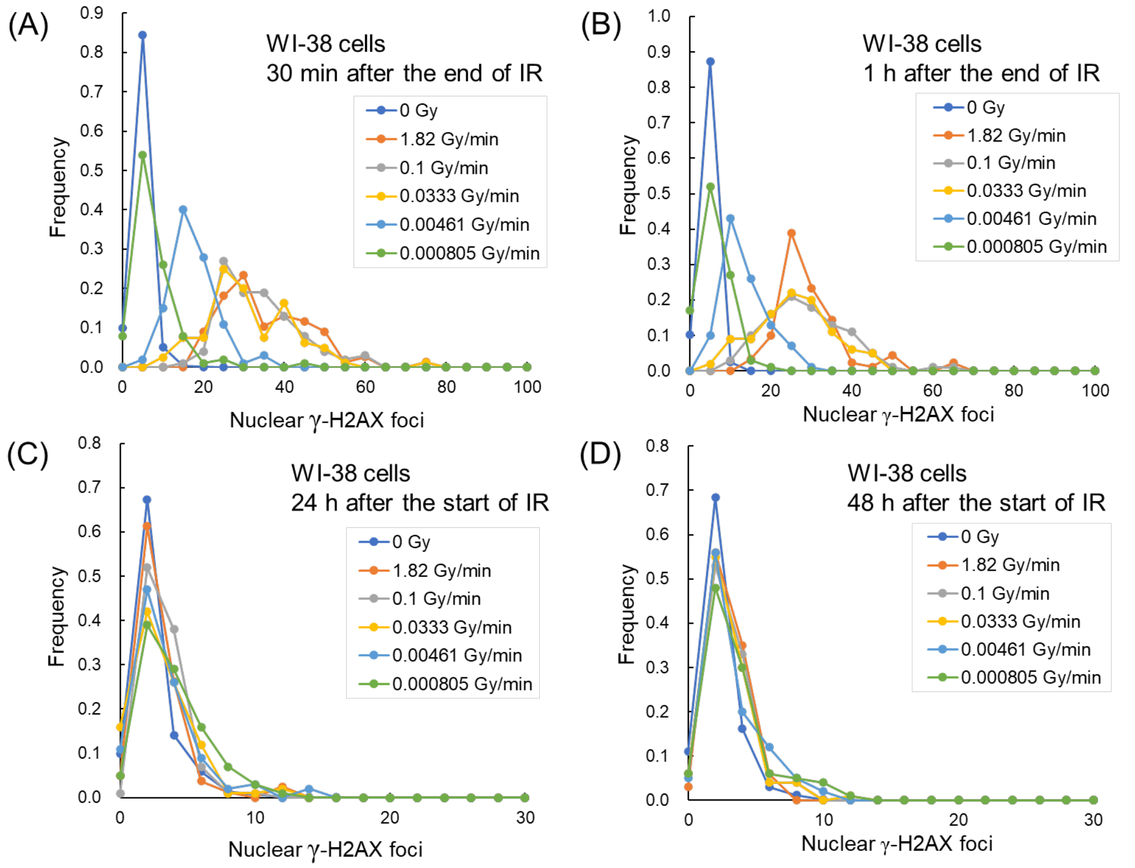

Figure S2. Distribution of nuclear γ-H2AX foci in WI-38 cells. (A) 30 min after the end of irradiation (IR), (B) 1 h after the end of ionizing irradiation (IR), (C) 24 h after the start of IR, and (D) 48 h after the start of IR. The nuclear γ-H2AX foci increased immediately after irradiation (i.e., 30 min after the end of IR) and decreased gradually after the irradiation, to the same level as the non-irradiated group 24 h and 48 h after the start of IR.

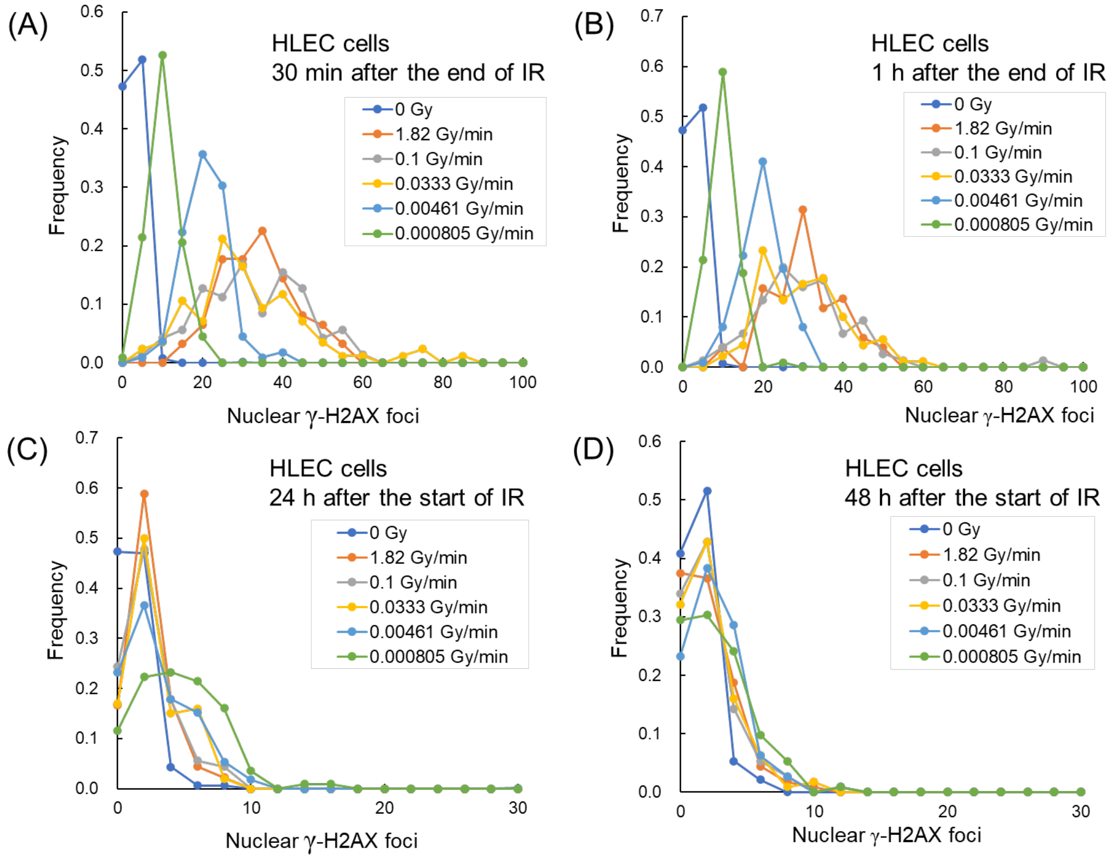

Figure S3. Distribution of nuclear γ-H2AX foci in HLEC. (A) 30 min after the end of IR, (B) 1 h after the end of IR, (C) 24 h after the start of IR, and (D) 48 h after the start of IR. The nuclear γ-H2AX foci induced immediately after irradiation (i.e., 30 min after the end of IR) were gradually repaired after the irradiation. However, the more residual DSBs were observed at 24 h after the start of IR. The residual DSBs sufficiently long after irradiation were more pronounced compared to the non-irradiated group, as the statistical evaluation was made in the main text (Fig. 4). The difference in the distribution between WI-38 and HLEC is due to the different repair rates: the DSB repair rates (c [h^{-1}] in the IMK model) of WI-38 and HLEC cells are 0.371 ± 0.038 and 0.309 ± 0.056 (h^{-1}), respectively. This suggests that the DSB repair is slower in HLECs than in WI-38 cells.

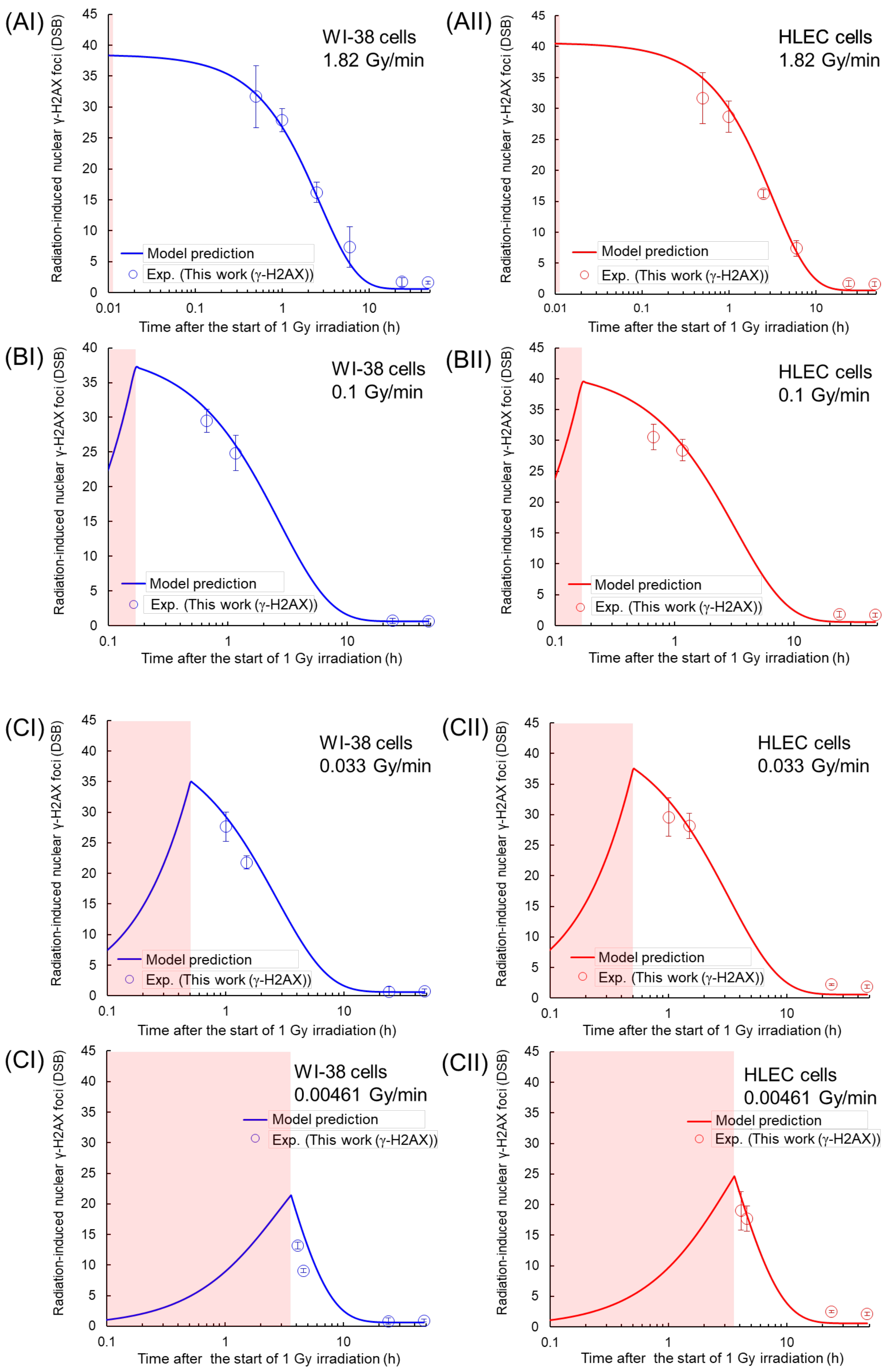
(AI)
WI-38 cells
1.82 Gy/min
Model prediction
Exp. (This work (γ-H2AX))
Radiation-induced nuclear γ-H2AX foci (DSB)
Time after the start of 1 Gy irradiation (h)
(AII)
HLEC cells
1.82 Gy/min
(BI)
WI-38 cells
0.1 Gy/min
(BII)
HLEC cells
0.1 Gy/min
(CI)
WI-38 cells
0.033 Gy/min
(CII)
HLEC cells
0.033 Gy/min
(CI)
WI-38 cells
0.00461 Gy/min
(CII)
HLEC cells
0.00461 Gy/min
Time after the start of 1 Gy irradiation (h)

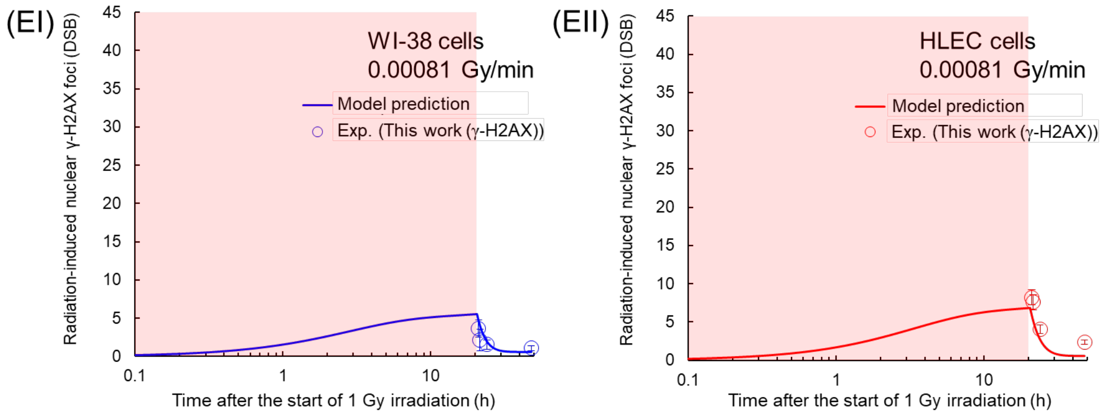

Figure S4. Comparison of time-dependent DSBs between the experiments and the model prediction. (A) radiation-induced nuclear foci at 1.82 Gy/min in WI-38 cells (I) and HLECs (II), (B) at 0.1 Gy/min in WI-38 cells (I) and HLECs (II), (C) at 0.033 Gy/min in WI-38 cells (I) and HLECs (II), (D) at 0.00461 Gy/min in WI-38 cells (I) and HLECs (II), (E) are at 0.00081 Gy/min in WI-38 cells (I) and HLECs (II). The symbol and solid line represent the experimental data and the prediction by the IMK model, respectively. The colored area the period of dose delivery. During the irradiation, the DNA damage induction competes with the repair, depending on the dose rates. As shown in Fig. S4, DSB kinetics estimated by the IMK model agree well with the experimental data.

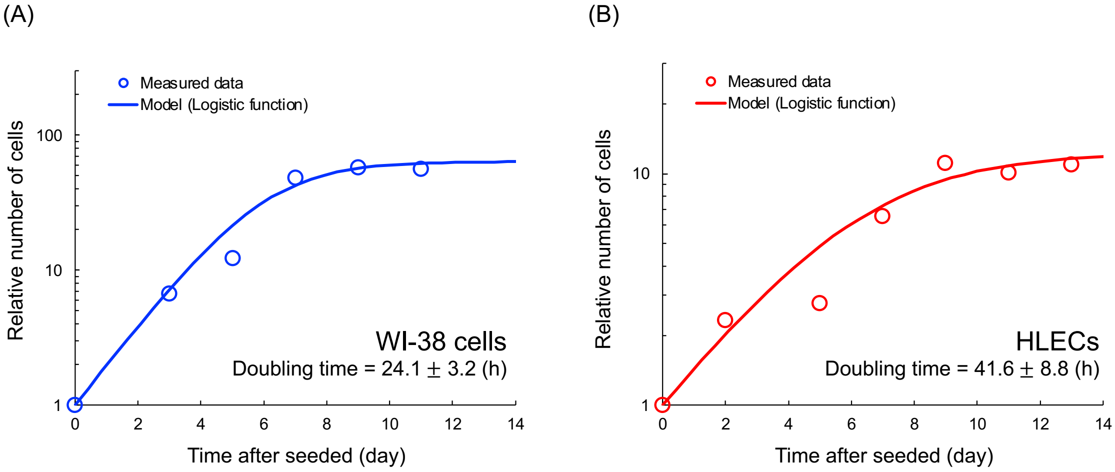

Figure S5. Growth curve and doubling time. (A) WI-38 cells, (B) HLECs. The symbol and solid line represent the experimental data and the prediction by the logistic function $[K/\{1+(K/N_0-1)\exp(-\mu t)\}]$, where K is the carrying capacity, N_0 is the initial number of cells at $t = 0$ (h) (i.e., $N_0 = 1$), and μ is the growth rate (h^{-1}). By fitting the function to the measured data with Markov chain Monte Carlo method, which is described in the main paper, the sets of the parameters (K, μ) for WI-38 and HLEC were (63.8 ± 11.1, 0.69 ± 0.09) and (12.4 ± 2.5, 0.40 ± 0.08), respectively. Doubling time was estimated as 24.1 ± 3.2 h in WI-38 and 41.6 ± 8.8 h in HLECs.

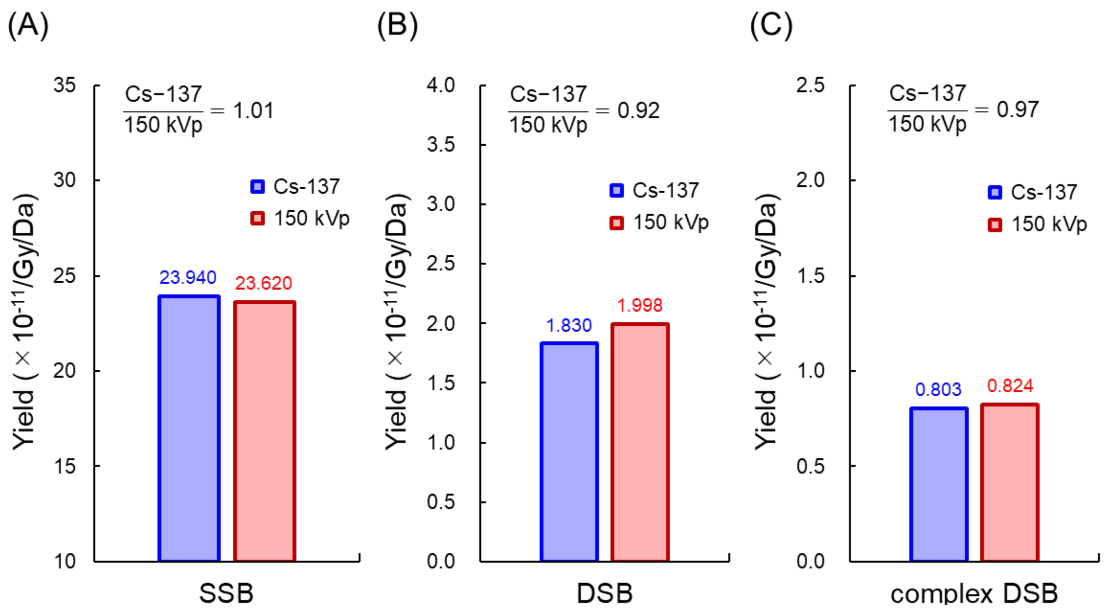

Figure S6. DNA damage yields estimated by the PHITS code. (A) the yields of single-strand break (SSB) for ^{137}Cs γ-rays and 150 kVp X-rays, (B) those of DNA double-strand break (DSB), and (C) those of complex DSB defined as the DSB coupled with additional strand breaks within 10-bp separation. The details of the estimation model are described.[3] From Fig. S6B, the relative yield of DSBs for ^{137}Cs γ-rays and that for 150 kVp X-rays was found to be 0.92, suggesting that the biological effects for DSBs for ^{137}Cs γ-rays are smaller compared to those for 150 kVp X-rays. Note that the PHITS estimation was validated previously.[4]

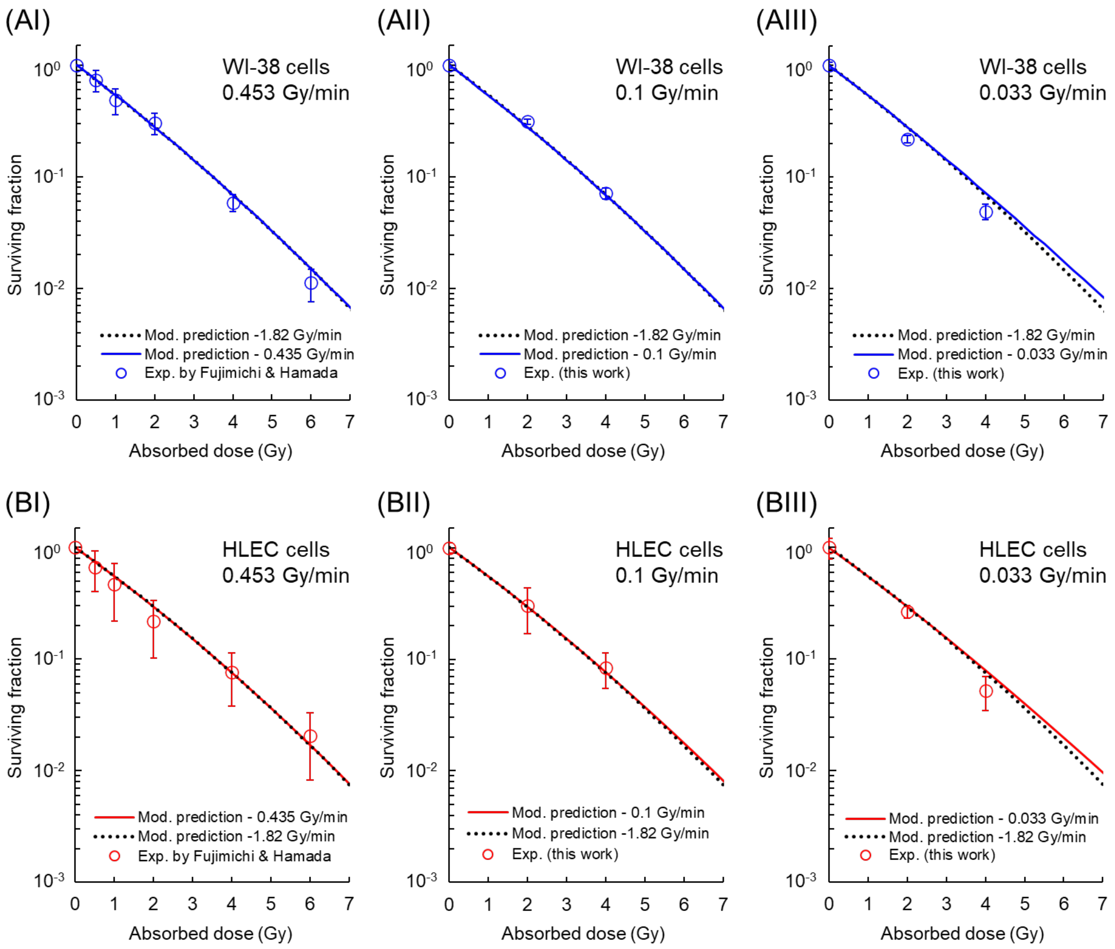

Figure S7. Dose-response curve of cell survival at various dose rates. (AI), (AII), and (AIII) are the curves of WI-38 cells at 0.453, 0.1, and 0.033 Gy/min, respectively. (BI), (BII), and (BIII) are the curves of HLECs at 0.453[5], 0.1, and 0.033 Gy/min, respectively. The symbol and solid line represent the experimental survival data and the prediction by the IMK model. The dotted line is the curve at 1.82 Gy/min. Comparing the curve at 1.82 Gy/min, there is little difference between cell survival at low and high dose rates. In the main text, the relationship between the absorbed dose rate and the surviving fraction 2 and 4 Gy after irradiation was depicted (see Fig. 6). From the results (Figs. 6 and S7), it can be concluded that there is no significant dose-rate dependence at 0.033–1.82 Gy/min. This supports the ICRP assumption of no dose rate effects.[6]

References

1. Matsuya, Y., *et al*. Investigation of dose-rate effects and cell-cycle distribution under protracted exposure to ionizing radiation for various dose-rates. *Sci. Rep.* **8**(1), 8287; 10.1038/s41598-018-26556-5 (2018).
2. Sato, T., *et al*. Recent improvements of the particle and heavy ion transport code system – PHITS version 3.33. *J. Nucl. Sci. Technol.* 10.1080/00223131.2023.2275736 (2023)
3. Matsuya, Y., *et al*. Track-structure mode in Particle and Heavy Ion Transport code System (PHITS): application to radiobiological research. *Int. J. Radiat. Biol.* **98** (2), 148–157 (2022).
4. Matsuya, Y., *et al*. Modeling of yield estimation for DNA strand breaks based on Monte Carlo simulations of electron track structure in liquid water. *J. Appl. Phys.* **126**, 124701 (2019).

5. Fujimichi Y, Hamada N. Ionizing Irradiation Not Only Inactivates Clonogenic Potential in Primary Normal Human Diploid Lens Epithelial Cells but Also Stimulates Cell Proliferation in a Subset of This Population. *PLoS One* **9**(5), e98154; 10.1371/journal.pone.0098154 (2014).

6. Hamada, N. Ionizing radiation sensitivity of the ocular lens and its dose rate dependence. *Int. J. Radiat. Biol.* **93**, 1024–1034 (1024).
